# Stage‐Specific H3K14 and H3K23 Succinylation Orchestrates Insect Metamorphosis and Oogenesis

**DOI:** 10.1002/advs.75624

**Published:** 2026-05-10

**Authors:** Yu‐Pu Jing, Lunjie Li, Libin Yang, Lin‐Jie Zhang, Qiang Yan, Ci Zhang, Zanrong Wen, Guangze Yu, Qian Li, Shutang Zhou

**Affiliations:** ^1^ State Key Laboratory of Cotton Bio‐Breeding and Integrated Utilization School of Life Sciences Henan University Kaifeng China; ^2^ HLA Laboratory Henan Red Cross Blood Center Zhengzhou Henan China

**Keywords:** fecundity, GPCR signaling, histone succinylation, insect metamorphosis, PKC isoform, post‐translational modification

## Abstract

Histone lysine succinylation is a pivotal epigenetic modification for diverse biological processes, yet how it regulates insect metamorphosis and reproduction remains poorly understood. Using *Locusta migratoria* as the primary model, we report here that protein succinylation is the most abundant acylation in the fat body, a highly metabolically active tissue analogous to vertebrate liver and adipose tissue. Succinylation of histone H3 on lysine 14 (suc‐H3^K14^) is predominant in nymphs, whereas H3K23 succinylation (suc‐H3^K23^) is highly abundant in adults. H3^K14A^ mutation causes embryonic lethality, and H3^K23A^ mutants exhibit remarkably reduced fecundity. P300 and GCN5 serve as succinyltransferases catalyzing suc‐H3^K14^ and suc‐H3^K23^, respectively. While P300‐mediated suc‐H3^K14^ is promoted via the GPCR‐PLC‐LTCC‐PKCα cascade, GCN5‐triggered suc‐H3^K23^ is achieved through the GPCR‐PLC‐TTCC‐PKCε axis. Loss of *P300* function and inhibition of suc‐H3^K14^ lead to precocious metamorphosis. Knocking down *GCN5* and suppression of suc‐H3^K23^ result in blocked oogenesis. Cut&Tag‐seq reveals that suc‐H3^K14^ and suc‐H3^K23^ target the genes regulating metamorphosis and vitellogenesis, respectively. Furthermore, suc‐H3^K14^‐repressed precocious metamorphosis and suc‐H3^K23^‐stimulated reproduction are evolutionarily conserved across divergent insect orders. The study significantly advances our understanding of how stage‐specific H3K succinylations coordinate insect metamorphosis and oogenesis, filling a gap in the epigenetic regulation of key life‐history traits.

## Graphical abstract

Stage‐specific succinylation of histone H3 at lysine 14 and 23 differentially controls insect metamorphosis and oogenesis through distinct GPCR‐PKC signaling cascades, revealing an evolutionarily conserved epigenetic mechanism that coordinates key life‐history traits.

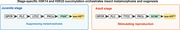

## 1 Introduction

Histone post‐translational modifications (PTMs) play a crucial role in the regulation of chromatin‐based processes and diverse biological functions such as gene expression, signal transduction, cellular metabolism, and organismal development [1]. Among the amino acids targeted by PTMs, lysine residues are most frequently post‐translationally modified. Compared with other lysine PTMs, succinylation appears to have more pronounced effects on histone structure and cellular functions because succinylation reverses the charge of lysine residues from positive to negative and adds a relatively larger structural moiety [2, 3, 4]. As a key epigenetic determinant of chromatin dynamics and gene transcription, histone lysine succinylation has been linked to a variety of physiological, pathophysiological, and tumorigenic processes [5]. E1A binding protein P300 and its paralogue CREB‐binding protein (CBP), lysine acetyltransferase 2A (KAT2A; also known as General Control Non‐Derepressible 5, GCN5), and histone acetyltransferase 1 (HAT1) can function as histone succinyltransferases. P300 catalyzes succinylation of histone H3 on K122 (H3^K122^), destabilizing nucleosomes and enhancing gene transcription [6]. P300 also mediates H3^K23^ succinylation to promote the expression of target genes in the colon cancer cells [7]. GCN5 succinylates H3^K79^ for erythropoiesis, diabetic nephropathy pathogenesis, and tumor development [8, 9, 10]. HAT1 modulates the succinylation of H3^K122^, contributing to epigenetic regulation and gene expression in the HepG2 cells [11]. Despite the advances of histone succinylation in vertebrates, little is known about the regulatory role of histone succinylation in insect metamorphosis and reproduction.

Insects are the most diverse animal group on Earth, partially owing to their metamorphosis and high fecundity, two extraordinary biological traits and highly successful strategies for environmental adaptation and biodiversity. The sesquiterpenoid juvenile hormone (JH) acts as both an anti‐metamorphic hormone that suppresses precocious metamorphosis in the larval stage [12, 13], and a gonadotrophic hormone stimulating reproductive aspects in adulthood [14]. Recent studies have shown that JH modulates protein PTMs via diverse signaling cascades to regulate insect metamorphosis and reproduction. For example, JH activates the GPCR‐PLC‐PKC cascade to phosphorylate the pupal‐specifier Broad‐complex, thereby inhibiting larval metamorphosis of *Helicoverpa armigera* [15]. JH acts via the RTK‐PI3K‐Akt axis to phosphorylate Serine/arginine‐rich (pre‐mRNA) splicing factor (SRSF), which facilitates the blood‐induced vitellogenesis of *Aedes aegypti* [16]. In *Locust migratoria*, *Periplaneta americana*, and *H. armigera*, JH activates the GPCR‐PLC‐PKCι pathway to trigger Vitellogenin receptor (VgR) phosphorylation, thus promoting vitellogenin (Vg) endocytosis into the maturing oocytes [17]. In *Drosophila melanogaster* and *Bombyx mori*, JH downregulates H3^K27^ trimethylation (H3^K27me3^), which inhibits *Hairy* transcription in the prothoracic gland and prevents the larval‐pupal transition [18]. JH also regulates the expression of *Histone deacetylase1* (*HDAC1*) and *HDAC3* for metamorphic modulation in *Tribolium castaneum* and *A. aegypti* [19]. However, the role of histone succinylation in JH‐regulated insect metamorphosis and reproduction remains unknown.

Functional screening of H3 lysine residues in *D. melanogaster* by CRISPR/Cas9‐mediated alanine‐substitution demonstrated that mutation on H3K4, H3K9, H3K14, H3K27, H3K37, H3K36, and H3K79 caused lethality, and H3K18, H3K23, H3K56, and H3K64 mutants conferred defects in viability and fertility, but their acylation types were not determined [20]. The migratory locust *L. migratoria*, a worldwide destructive insect pest, has been a favorite model in studying JH‐regulated metamorphosis and reproduction. In this study, by using *L. migratoria* as the primary model organism, we revealed that succinylation was the most abundant lysine acylation in the fat body, with stage‐specific enrichment of H3^K14^ succinylation (suc‐H3^K14^) in the penultimate instar nymphs and H3^K23^ succinylation (suc‐H3^K23^) in the vitellogenic adults. We further demonstrated that suc‐H3^K14^ was catalyzed by P300 through JH‐mediated GPCR‐PLC‐LTCC‐PKCα pathway in nymphs, and suc‐H3^K23^ was promoted by GCN5 via the GPCR‐PLC‐TTCC‐PKCε cascade in adults. While knockdown of *P300* and inhibition of suc‐H3^K14^ resulted in precocious metamorphosis, depletion of *GCN5* and suppression of suc‐H3^K23^ led to arrested oogenesis. Moreover, these regulatory modules are evolutionarily conserved across diverse insect species.

## 2 Results

### 2.1 Succinylation is Among the Most Abundant Lysine Acylations in the Fat Body

**FIGURE 1:**
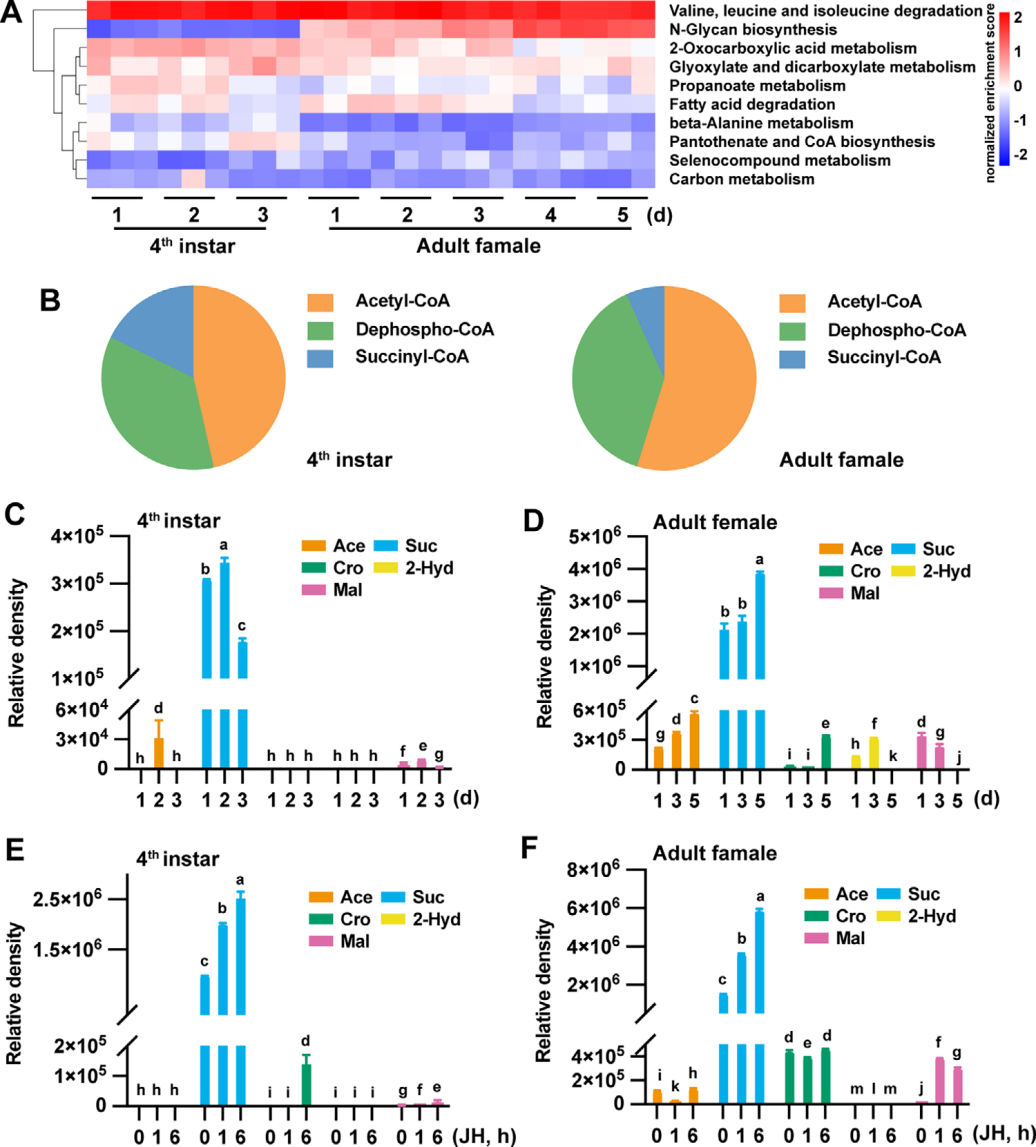
Abundant protein succinylation in the fat body. (A) Top 10 KEGG metabolic pathways from ssGSEA analysis of RNA‐seq on the fourth instar nymphs and adult females of *L. migratoria*. (B) Relative abundance of succinyl‐CoA, acetyl‐CoA, and dephospho‐CoA measured by LC‐MS/MS in the fat body of fourth instar nymphs (day 2) and adult females (day 4). (C, D) Relative abundance of lysine acylations in the fourth instar nymphs (*C*) and adult females (*D*). (E, F) Relative levels of lysine acylation in the fourth instar nymphs (day 1, *E*) and adult females (day 1, *F*) treated with JH for 1–6 h. Ace, acetylation; Suc, succinylation; Cro, crotonylation; 2‐Hyd, 2‐hydroxy‐isobutyrylation; Mal, malonylation. Different letters indicate a significant difference at *p* < 0.05, *n* = 3.

The fat body is a central hub for nutritional storage, energy mobilization, and Vg synthesis during insect metamorphosis and reproduction [21]. We initially performed RNA‐seq of fat bodies isolated from the penultimate fourth instar nymphs and adult females of *L. migratoria*. Single‐sample gene set enrichment analysis (ssGSEA) identified 79 significantly enriched KEGG pathways associated with metabolism, with the valine, leucine, and isoleucine metabolism pathway ranking at the top in both nymphs and adults (Figure 1A; Figure S1). Valine, leucine, and isoleucine produce acetyl‐coenzyme A (acetyl‐CoA) and propionyl‐CoA, which are further converted to succinyl‐CoA [22, 23]. LC‐MS/MS analysis of coenzymes revealed that succinyl‐CoA was among the top three CoAs in the fat body, following acetyl‐CoA and dephospho‐CoA (Figure 1B).

Given that the intracellular CoA is associated with lysine acylation [24], we next conducted capillary‐based western blotting to quantify lysine acylation in the fat body. Compared to lysine acetylation (Ace), crotonylation (Cro), 2‐hydroxy‐isobutyrylation (2‐Hyd), and malonylation (Mal), much stronger signals were observed with succinylation (Figure S2A,B). Statistically, the abundance of lysine succinylation was > 8.3‐fold and > 4.2‐fold higher than the other four acylations in the nymphs and adults, respectively (Figure 1C,D). Interestingly, the abundance of lysine succinylation decreased in late fourth instar nymphs (day 3) compared to that on day 1–2 (Figure 1C; Figure S2A). In the adult stage, lysine succinylation increased in the fat body of vitellogenic females (day 5) compared to previtellogenic females (day 1–3) (Figure 1D; Figure S2B). As JH titers are low at nymphal molting but high in the vitellogenic period [25, 26], the variation of lysine succinylation appeared to correlate with JH titers. We therefore evaluated the responsiveness of lysine acylation to JH. Application of JH significantly increased succinylation in the fat body of both nymphs and adults, in a time‐dependent manner (Figure 1E,F; Figure S2C,D). However, such patterns were not observed with lysine acetylation, crotonylation, 2‐hydroxy‐isobutyrylation, or malonylation (Figure 1E,F; Figure S2C,D). The data indicate that succinylation is among the most abundant lysine acylations in the fat body and is inducible by JH.

### 2.2 Suc‐H3^K14^ and Suc‐H3^K23^ Exhibit Stage‐Specific Profiling

**FIGURE 2:**
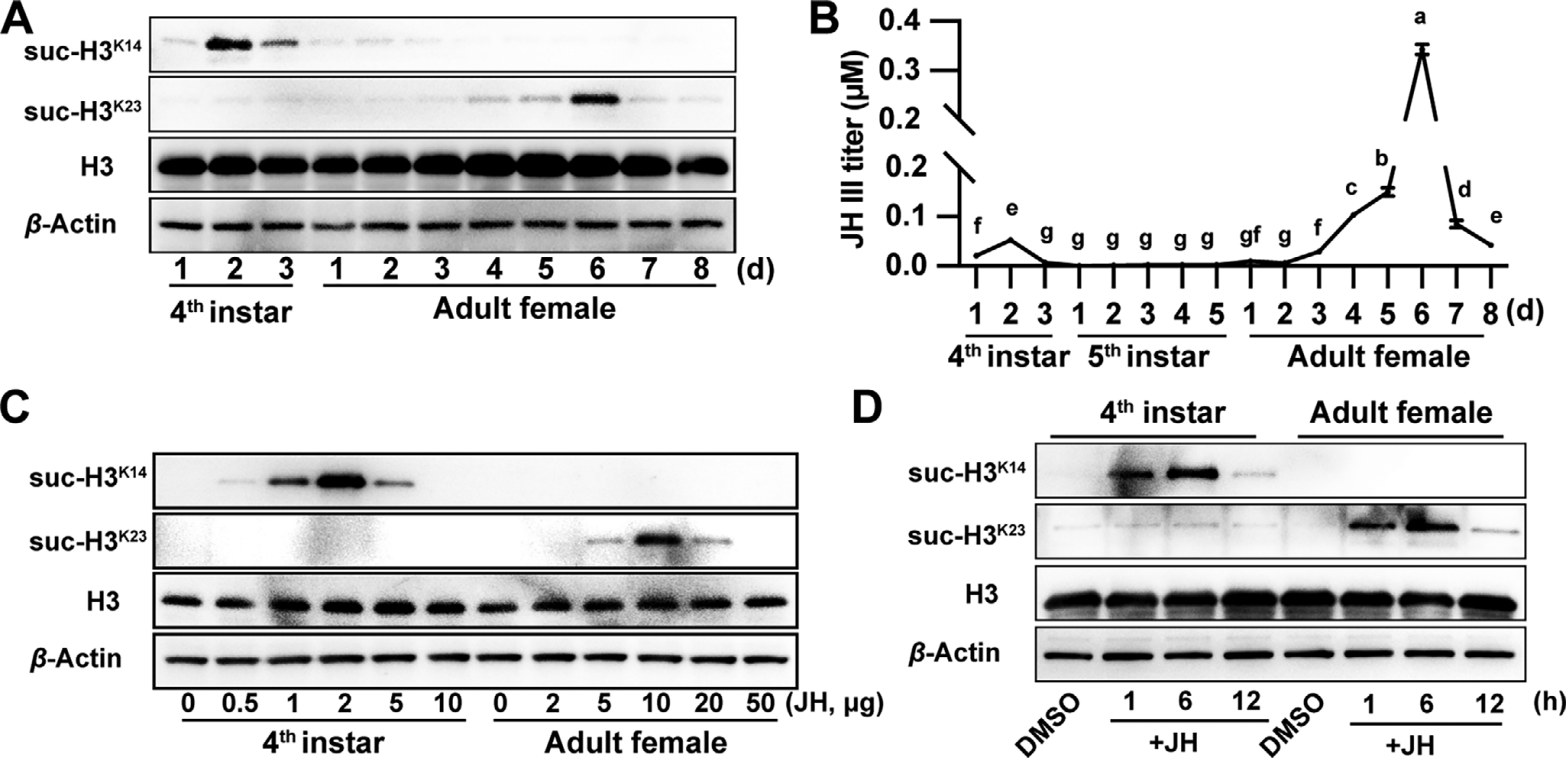
Stage‐specific responsiveness of suc‐H3^K14^ and suc‐H3^K23^ to JH. (A) Temporal abundance of suc‐H3^K14^ and suc‐H3^K23^ in the fat body of fourth instar nymphs (days 1–3) and adult females (days 1–8 post eclosion) of *L. migratoria*. (B) Dynamics of JH titers in the hemolymph of fourth instar nymphs to adults. Different letters indicate a significant difference at *p* < 0.05, *n* = 3. (C) Dose response of suc‐H3^K14^ and suc‐H3^K23^ to JH for 1 h in the fourth instar nymphs (day 1) and adult females (day 3). (D) Time course of suc‐H3^K14^ and suc‐H3^K23^ responsiveness to JH in the fourth instar nymphs (day 1) and adult females (day 3) at 2 µg and 10 µg, respectively.

Considering the functional importance of histone succinylation in the nucleosome structure and genome‐wide transcription, we focused our study on histone lysine succinylation. To identify the succinylated histone lysine residues, we performed succinylome profiling and LC‐MS/MS of fat body proteins extracted from mid fourth instar nymphs (day 2) and early vitellogenic females (4 days post adult emergence) of *L. migratoria*. Four succinylation sites of H3K residues were identified, including H3K14 (suc‐H3^K14^), H3K23 (suc‐H3^K23^), H3K79 (suc‐H3^K79^), and H3K122 (suc‐H3^K122^) (Figure S3A–D). Developmental profiling revealed that suc‐H3^K14^ was highly abundant in the penultimate fourth instar nymphs but at extremely low levels in the adults (Figure 2A). Conversely, the level of suc‐H3^K23^ was considerably low in the nymphs, gradually elevated in the previtellogenic phase, and rose to a peak at the early vitellogenic period (Figure 2A). However, the abundance of suc‐H3^K79^ decreased in late fourth instar nymphs and vitellogenic adults, while suc‐H3^K122^ remained at constant levels (Figure S3E,F). The data indicate that suc‐H3^K14^ predominantly takes place in the penultimate instar nymphs, whereas suc‐H3^K23^ is rich in the vitellogenic adults.

Since JH plays an essential role in inhibiting precocious metamorphosis and stimulating adult reproduction, we measured JH titers in the hemolymph of locusts from the fourth instar to adults. As shown in Figure 2B, the JH titer was high in mid fourth instar and then significantly dropped in late fourth instar. In adulthood, JH titer increased significantly in the previtellogenic period, reached a peak at mid vitellogenic stage, and then declined at late vitellogenic stage (Figure 2B). Overall, JH titer was significantly higher in the adults than the nymphs (Figure 2B), suggesting a variable effect of JH on suc‐H3^K14^ and suc‐H3^K23^ modifications. Dose‐response experiments demonstrated that JH at low dose (2 µg) induced the highest levels of suc‐H3^K14^ in the nymphs, while a high dose of JH (10 µg) maximized suc‐H3^K23^ in the adults (Figure 2C). Time‐course analysis demonstrated that JH treatment led to increased levels of suc‐H3^K14^ and suc‐H3^K23^ within 1–6 h, followed by a decline (Figure 2D). Taken together, the above results suggest that suc‐H3^K14^ and suc‐H3^K23^ are JH‐responsive and confer stage‐specific functions.

### 2.3 Suc‐H3^K14^ and Suc‐H3^K23^ Differentially Regulate Insect Metamorphosis and Reproduction

**FIGURE 3:**
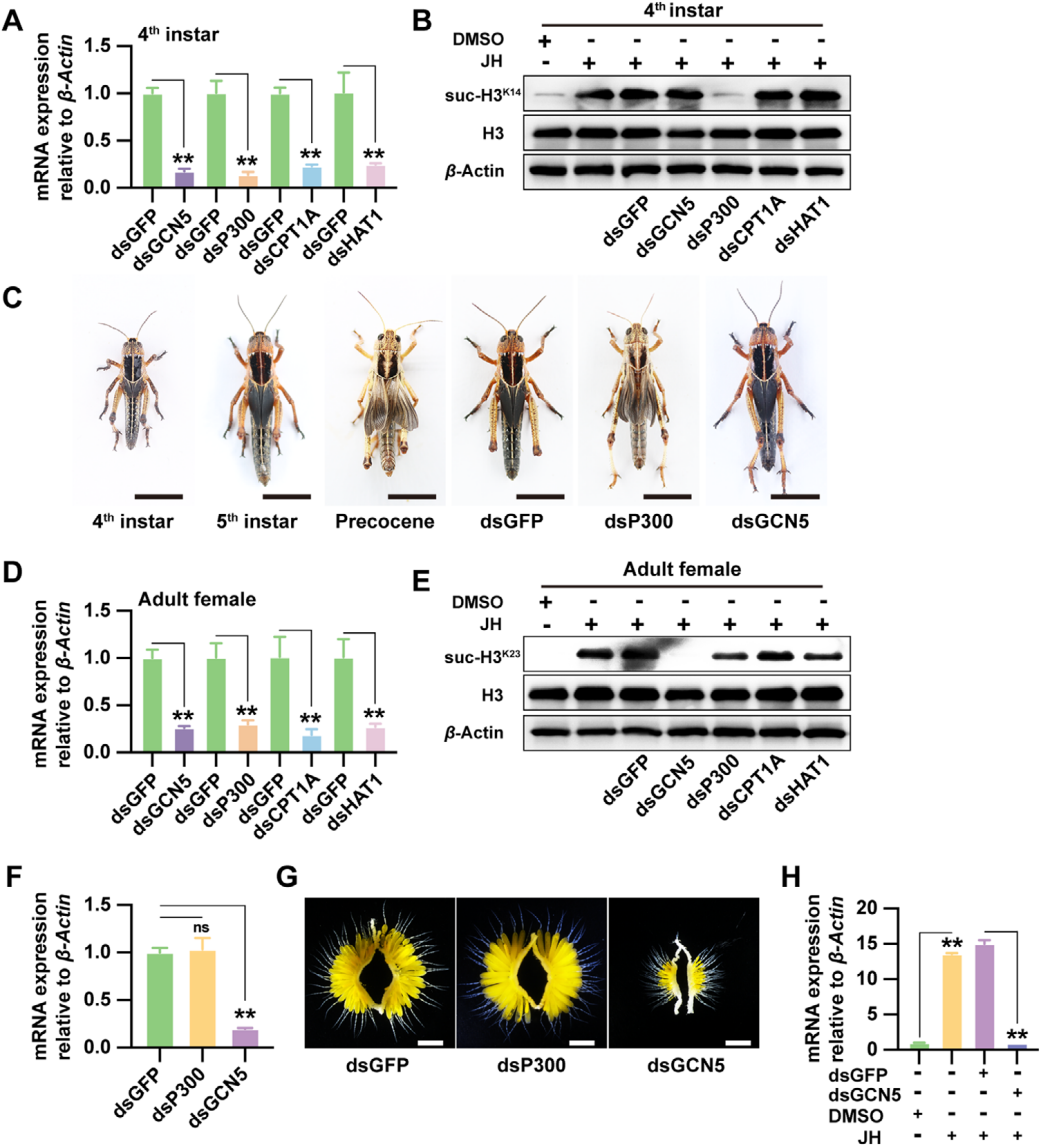
Differential role of suc‐H3^K14^ and suc‐H3^K23^ in the metamorphosis and reproduction. (A) Knockdown efficiency of lysine succinyltransferases (*P300*, *GCN5*, *CPT1A, and HAT1*) in the fat body of fourth instar nymphs of *L. migratoria*. ^**^, *p* < 0.01; *n* = 3. (B) Effect of succinyltransferase knockdown on JH‐stimulated suc‐H3^K14^ in the fat body of fourth instar nymphs. DMSO, solvent control. (C) Representative ecdysis phenotypes of fourth instar nymphs treated with precocene, dsGFP, dsP300, or dsGCN5. fifth instar, normal final fifth instar nymph. Scale bars, 1 cm. (D) Knockdown efficiency of succinyltransferases in the fat body of adult females. ^**^, *p* < 0.01; *n* = 3. (E) Effect of succinyltransferase knockdown on JH‐stimulated suc‐H3^K23^ in the fat body of adult females. (F) Effect of *P300* or *GCN5* knockdown on *Vg* expression in the fat body of adult females (day 6). ns, no significant difference; ^**^, *p* < 0.01; *n* = 3. (G) Representative ovarian phenotypes of 6‐day‐old adult females subjected to *P300* or *GCN5* RNAi. Scale bars, 5 mm. (H) Effect of *GCN5* knockdown on JH‐induced *Vg* expression in the fat body of adult females (day 3). ^**^, *p* < 0.01; *n* = 3.

Previous studies have shown the involvement of P300/CBP, GCN5, HAT1, and carnitine palmitoyltransferase 1A (CPT1A) in histone succinylation [9, 27]. We next determined which succinyltransferases trigger suc‐H3^K14^ and suc‐H3^K23^. For suc‐H3^K14^ in the nymphs, only *P300* knockdown suppressed JH‐induced suc‐H3^K14^, whereas depletion of *GCN5*, *CPT1A*, or *HAT1* had no obvious impact on JH‐induced suc‐H3^K14^ (Figure 3A,B). In the penultimate fourth instar nymphs, loss of *P300* function and consequent inhibition of suc‐H3^K14^ (Figure S4A,B) resulted in precocious metamorphosis to adults, including adult‐type body pigmentation, wing pad expansion, and developed genitalia, resembling to the phenotypes caused by deprival of endogenous JH (Figure 3C). The developmental duration from the fourth instar nymph to the next stage between the *P300*‐depleted and dsGFP groups had no significant difference (Figure S4C). Notably, the penultimate fourth instar nymphs subjected to *GCN5* RNAi that had no impact on suc‐H3^K14^ normally molted to the final fifth instar, mimicking the dsGFP controls (Figure 3C; Figure S4A–C). For suc‐H3^K23^ in the adults, knockdown of *GCN5* but not *P300*, *CPT1A* or *HAT1* inhibited JH‐induced suc‐H3^K23^ (Figure 3D,E). *GCN5* knockdown and concomitant inhibition of suc‐H3^K23^ (Figure S4C,D) led to significantly reduced *Vg* expression in the fat body (Figure 3F), accompanied by arrested ovarian development and blocked oocyte maturation (Figure 3G). Moreover, *GCN5* knockdown‐mediated inhibition of suc‐H3^K23^ precluded JH induction of *Vg* expression in the fat body of adult females (Figure 3H). By contrast, no defective phenotypes were observed with *Vg* expression, ovarian development or oocyte maturation in the adult females subjected to *P300* RNAi that had no effect on suc‐H3^K23^ (Figure 3F,G; Figure S4D,E). *GCN5* knockdown had no significant influence on the developmental duration from adult ecdysis to first oviposition (Figure S4F), but remarkably reduced the number of laid eggs (Figure S4G). In contrast, knocking down *P300* had no significant effect on laid egg number (Figure S4G). While laid egg number were remarkably decreased in *GCN5*‐depleted groups, the egg hatching rate was not significantly affected (Figure S4H). Taken together, the above data indicate that P300 catalyzes suc‐H3^K14^ in regulating nymphal metamorphosis, and GCN5 promotes suc‐H3^K23^ in modulating adult reproduction.

As H3^K14^ and H3^K23^ can be post‐translationally modified by multiple acylations catalyzed by acetyltransferases, including P300, GCN5, CPT1A, and HAT1 [28, 29, 30, 31], we further investigated the effect of *P300*, *GCN5*, *CPT1A*, and *HAT1* RNAi on other types of acylation at H3^K14^ and H3^K23^. As shown in Figure S5, though H3^K14^ acetylation (Ace‐H3^K14^), crotonylation (Cro‐H3^K14^), and 2‐hydroxy‐isobutyrylation (2‐Hyd‐H3^K14^) were observed in the fourth instar nymphs, none was induced by JH treatment. Notably, *P300*, *GCN5*, *CPT1A* and *HAT1* knockdown had no significant effect on the abundance of Ace‐H3^K14^, Cro‐H3^K14^ or 2‐Hyd‐H3^K14^ (Figure S5A,a^1^‐a^3^). H3^K14^ malonylation (Mal‐H3^K14^) was not examined due to the unavailability of a commercial antibody. In the case of H3^K23^ in the adults, though its acetylation (Ace‐H3^K23^), crotonylation (Cro‐H3^K23^), 2‐hydroxy‐isobutyrylation (2‐Hyd‐H3^K23^), and malonylation (Mal‐H3^K23^) were also detected, neither JH treatment nor depletion of genes coding for these four acyltransferases had a conspicuous effect on Ace‐H3^K23^, Cro‐H3^K23^, 2‐Hyd‐H3^K23^ or Mal‐H3^K23^ (Figure S5B,b^1^‐b^4^). These results suggest that P300 primarily catalyzes suc‐H3^K14^ in the nymphs, and GCN5 primarily promotes suc‐H3^K23^ in the adults.

**FIGURE 4:**
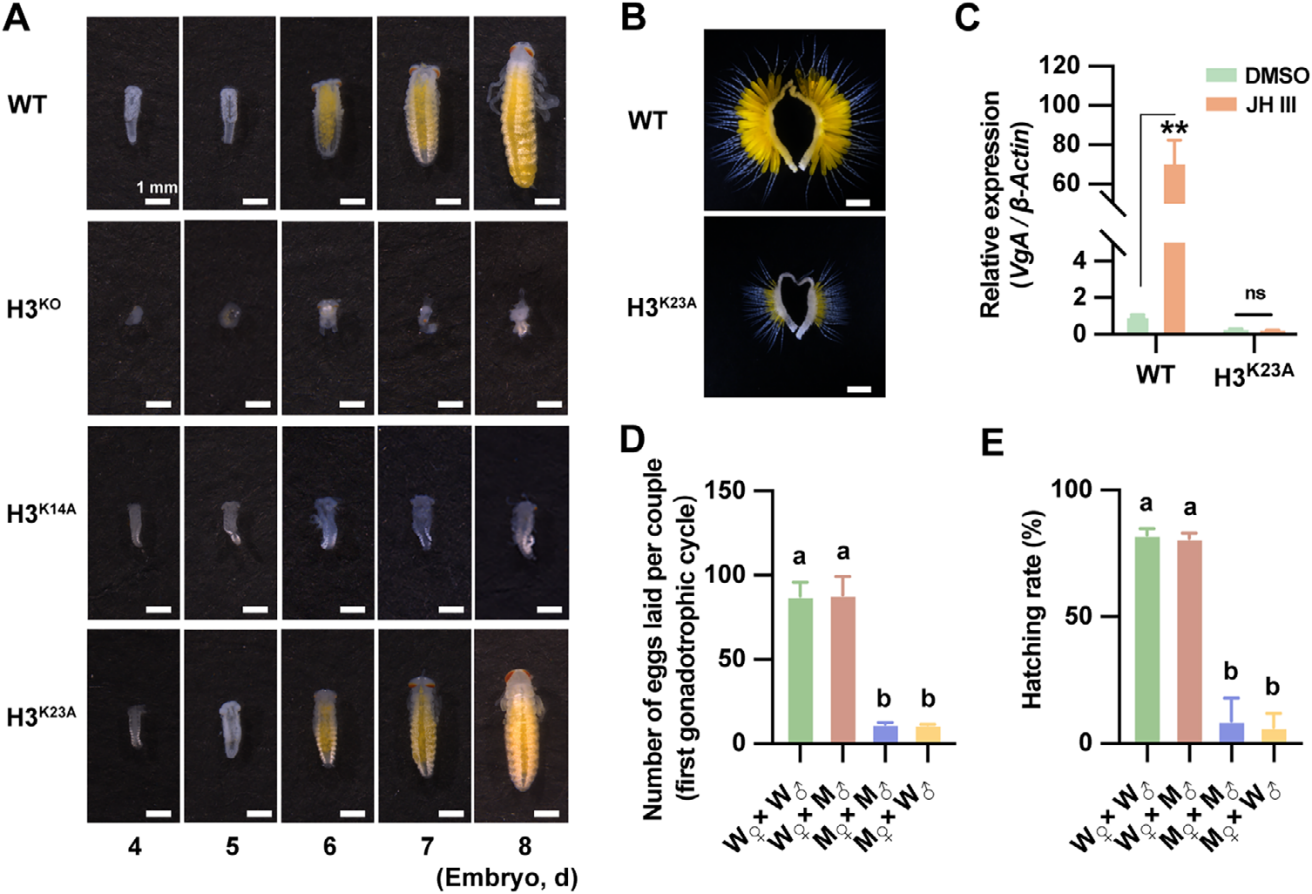
Defective phenotypes of H3^K14A^ and H3^K23A^ mutant females. (A) Representative embryonic phenotypes of H3^KO^, H3^K14A^, and H3^K23A^ mutants vs. wildtype (WT) locusts. Scale bars: 1 mm. (B) Representative ovarian phenotypes of H3^K23A^ mutants (day 6). Scale bars: 2 mm. (C) Relative expression levels of *Vg* in the fat body of 6‐day‐old H3^K23A^ mutants treated with JH. ^**^, *p* < 0.01; ns, no significant difference; *n* = 3. (D) Number of eggs laid by H3^K23A^ vs. WT females mated with WT or H3^K23A^ males in the first gonadotrophic cycle. (E) Hatching rate of eggs laid by H3^K23A^ mutant vs. wild‐type adult females. M, H3^K23A^ mutants; W, wildtypes. Different letters indicate a significant difference at *p* < 0.05, *n* = 3.

To further define the role of suc‐H3^K14^ and suc‐H3^K23^ in insect metamorphosis and reproduction, we employed CRISPR/Cas9 to generate H3 knockout (H3^KO^) as well as alanine‐substituted H3^K14A^ and H3^K23A^ mutants of *L. migratoria* (Figure S6). H3^KO^ and H3^K14A^ mutants were lethal in embryogenic development (Figure 4A). H3^K23A^ mutants were capable of developing successfully from the embryos to adults (Figure 4A), but exhibited arrested ovarian development and inhibited oocyte maturation (Figure 4B). Furthermore, the induction of *Vg* by JH was eliminated in H3^K23A^ mutants (Figure 4C). The fertility of wildtype females crossing with H3^K23A^ mutant males had no significant difference from that of mating with wildtype males (Figure 4D,E). However, H3^K23A^ mutant females mating with either wildtype or H3^K23A^ mutant males laid remarkably fewer eggs than the wildtype females, along with a significant reduction of hatching rates (Figure 4D,E). Collectively, the results suggest that suc‐H3^K14^ plays a crucial role in metamorphic transition, and suc‐H3^K23^ is essential for oogenesis.

**FIGURE 5:**
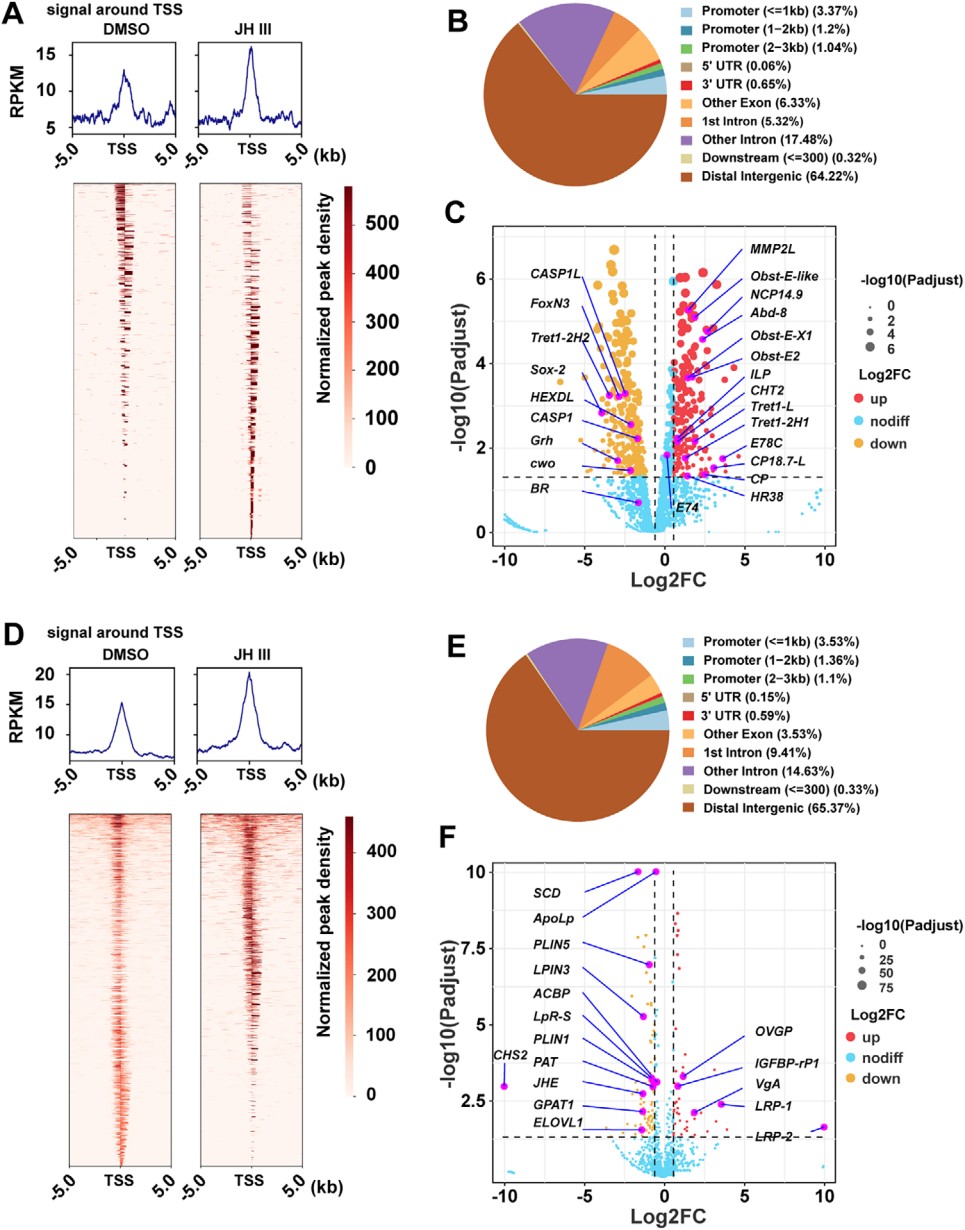
CUT&Tag‐seq analysis of suc‐H3^K14^ and suc‐H3^K23^. (A) Binding profiles of suc‐H3^K14^ in the fat body of fourth instar nymphs of *L. migratoria* treated with JH or DMSO. RPKM, reads per kilobase per million mapped reads; TSS, transcription start site. (B) Pan chart showing genomic distribution of suc‐H3^K14^ peaks. (C) Volcano plot showing DEGs targeted by suc‐H3^K14^ in the fat body instar nymphs under JH vs. DMSO treatment. (D) Binding profiles of suc‐H3^K23^ in the fat body of adult females treated with JH or DMSO. (E) Genomic distribution of suc‐H3^K23^ peaks. (F) DEGs regulated by suc‐H3^K23^ in the fat body of adult females under JH vs. DMSO treatment.

We next performed Cut&Tag‐seq and RNA‐seq on the fat bodies of early fourth instar nymphs (suc‐H3^K14^ focus) and previtellogenic adults (suc‐H3^K23^ focus) of locusts treated with JH vs. DMSO. In the nymphs, RNA‐seq identified 14,880 differentially expressed genes (DEGs), and Cut&Tag‐seq revealed 4,262 genes with JH‐induced suc‐H3^K14^ enrichments and 21,340 peaks (Figure 5A). Among these peaks, 5.27% were localized to the promoters, 31.2% to introns, 28.5% to exons, and 35.03% to intergenic regions (Figure 5B). Integrative analysis of Cut&Tag‐seq and RNA‐seq datasets revealed that 190 JH‐upregulated and 299 JH‐downregulated genes were enriched with suc‐H3^K14^ in the fourth instar nymphs. KEGG pathway enrichment analysis showed that these DEGs are classified into multiple functional categories, with carbohydrate metabolism, amino acid metabolism, and lipid metabolism as the top‐enriched pathways (Figure S7A,B). The suc‐H3^K14^‐targeted and JH‐responsive DEGs include the genes involved in cuticle formation such as *nymph cuticular protein* (*NCP14.9*), *endocuticle structural glycoprotein SgAbd‐8* (*Abd‐8*), *cuticle protein 18.7‐like* (*CP18.7‐L*) and *cuticle protein* (*CP*), as well as chitin and cuticle matrix remodeling such as *obstructor‐E‐like* (*Obst‐E‐like*), *obstructor‐E isoform X1* (*Obst‐E1*), *obstructor E2* (*Obst‐E2*), and *probable chitinase 2* (*CHT2*) [32, 33, 34, 35] (Figure 5C). DEGs associated with sugar transport and metabolism, including *Tret1‐like* (*Tret‐L*), *Tret1‐2 homolog* 2 (*Tret1‐2H2*), *Tret1‐2 homolog 1* (*Tret1‐2H1*), and *hexosaminidase D‐like* (*HEXDL*) [36, 37] were targeted by suc‐H3^K14^ (Figure 5C). Notably, DEGs involved in metamorphosis, such as *ecdysone‐induced protein 78C* (*E78C*), *ecdysone‐induced protein 74* (*E74*)*, hormone receptor 38* (*HR38*), *Broad‐complex* (*BR*), and development, such as *grainyhead* (*Grh*), *forkhead box protein N3* (*FoxN3*), *SRY‐box transcription factor 2* (*Sox‐2*), *clockwork orange* (*cwo*), *insulin‐like growth peptide 1* (*ILP1*), *matrix metalloproteinase‐2‐like* (*MMP2L*), and *caspase‐1* (*CASP1*), were enriched with suc‐H3^K14^ [38, 39, 40, 41, 42, 43] (Figure 5C). To validate the direct binding of suc‐H3^K14^ to these DEGs, we performed ChIP‐qPCR in the promoter regions indicated by Integrative Genomics Viewer (IGV) tracks (Figure S8A). JH treatment significantly altered the enrichment of suc‐H3^K14^ in the promoter regions of selected genes except *Obst‐E1* and *E74* (Figure S8B–X), suggesting that suc‐H3^K14^ directly regulates the transcription of those 21genes. qRT‐PCR validation showed that all 23 genes were expressed in response to JH (Figure S9), confirming the reliability of RNA‐seq.

In adults, RNA‐seq identified 11,391 DEGs, and Cut&Tag‐seq revealed 2,400 genes with JH‐induced suc‐H3^K23^ enrichments and 5,662 peaks (Figure 5D). Of these peaks, 6.03% were localized to promoters, 33.8% to introns, 26.1% to exons, and 34.07% to intergenic regions (Figure 5E). Integrative analysis of Cut&Tag‐seq and RNA‐seq datasets revealed that 36 JH‐upregulated and 65 JH‐downregulated genes were enriched with suc‐H3^K23^. KEGG pathway enrichment analysis showed that these genes belong to multiple functional categories, with metabolism and organismal systems as the top‐enriched pathways (Figure S10A,B). In the metabolism category, the highly enriched pathways include carbon metabolism, nucleotide (purine and pyrimidine) metabolism, sugar metabolism, and lipid metabolism (Figure S10B). The suc‐H3^K23^‐targeted and JH‐responsive DEGs include the genes involved in reproduction, such as *Vg*, *vitellogenin receptor‐like/LDL receptor‐related protein 1* (*LRP1*), *LRP2*, *juvenile hormone esterase* (*JHE*), *apolipophorin* (*ApoLp*), and *lipophorin receptor* (*LpR‐S*) [44, 45, 46] (Figure 5F). DEGs associated with lipid metabolism, such as *perilipin‐5* (*PLIN5*), *perilipin‐1* (*PLIN1*), *fatty acid elongase 1* (*ELOVL1*), *glycerol‐3‐phosphate acyltransferase 1* (*GPAT1*), *phosphatidate phosphatase LPIN* (*LPIN3*), and *acyl‐CoA‐binding protein* (*AcBP*) were targeted by suc‐H3^K23^ (Figure 5F). Additionally, DEGs involved in nutrient signaling, including *insulin‐like growth factor‐binding protein‐related protein 1* (*IGFBP‐rP1*), *proton‐coupled amino acid transporter* (*PAT*), glucose‐6‐phosphate exchanger SLC37A2‐like (*SLC37A2L*), and reproductive structural remodeling, such as *Oviduct‐specific glycoprotein* (*OVGP*), were enriched with suc‐H3^K23^ [47, 48, 49, 50] (Figure 5F). We also carried out ChIP‐qPCR to validate the direct binding of suc‐H3^K23^ to 16 DEGs in the promoter regions indicated by IGV tracks (Figure S11A). With the exception of *ELOVL1*, JH treatment led to significantly increased enrichment of suc‐H3^K23^ in the promoter regions of the other 15 genes (Figure S11B–Q), suggesting the direct regulation of suc‐H3^K23^ in these gene transcription. qRT‐PCR validation confirmed that all these genes were expressed in response to JH (Figure S12).

### 2.4 Suc‐H3^K14^ and Suc‐H3^K23^ are Evolutionarily Conserved Across Insect Orders

**FIGURE 6:**
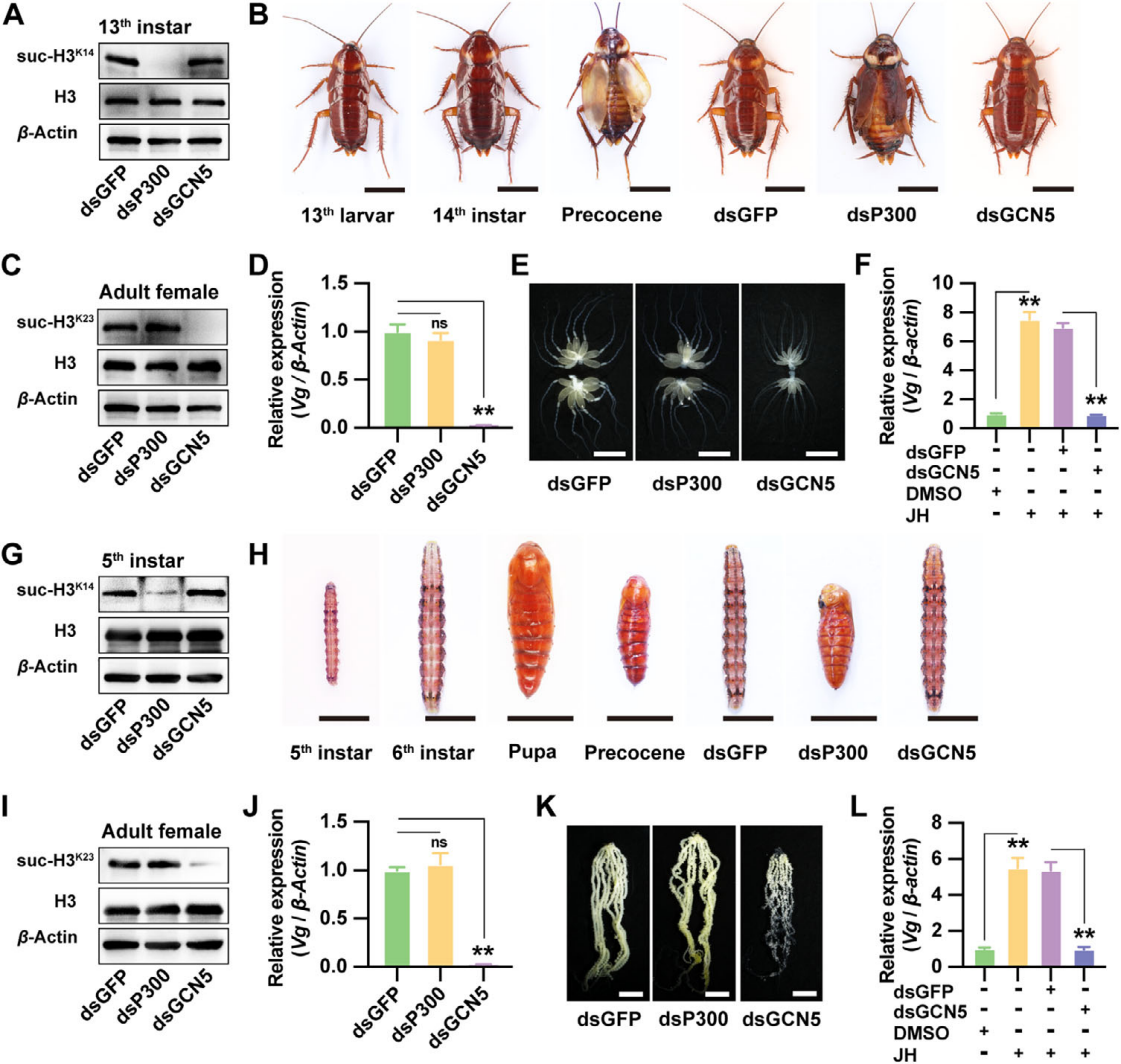
Evolutionary conservation of suc‐H3^K14^ and suc‐H3^K23^ in both hemimetabolous and holometabolous species. (A) P300‐mediated suc‐H3^K14^ in the penultimate 13th instar nymphs of *P. americana*. (B) Precocious metamorphosis of 13th instar nymphs caused by *P300* knockdown. Scale bars, 1 cm. (C) GCN5‐catalyzed suc‐H3^K23^ in the adult females. (D) Effect of *GCN5* knockdown on *Vg* expression in the vitellogenic females. ns, no significant difference; ^**^, *p* < 0.01; *n* = 3. (E) Defective ovarian phenotypes of vitellogenic females subjected to *GCN5* RNAi. Scale bars, 5 mm. (F) Effect of *GCN5* knockdown on JH‐induced *Vg* expression in the previtellogenic females. ^**^, *p* < 0.01; *n* = 3. (G) P300‐mediated suc‐H3^K14^ in the penultimate fifth instar larvae of *H. armigera*. (H) Precocious metamorphosis of fifth instar larvae caused by *P300* knockdown. Scale bars, 5 mm. (I) GCN5‐catalyzed suc‐H3^K23^ in the adult females. (J) Effect of *GCN5* knockdown on *Vg* expression. ns, no significant difference; ^**^, *p* < 0.01; *n* = 3. (K) Defective ovarian development of adult females subjected to *GCN5* RNAi. Scale bars, 5 mm. (L) Effect of *GCN5* knockdown on JH‐induced *Vg* expression. DMSO, solvent control. ^**^, *p* < 0.01; *n* = 3.

In hemimetabolous *P. americana*, suc‐H3^K14^ was also restrained by *P300* knockdown in the nymphs (Figure 6A; Figure S13A). Depletion of *P300* and consequent impairment of suc‐H3^K14^ resulted in the penultimate 13th instar nymphs precociously metamorphosing to adults, matching the effects of JH deficiency (Figure 6B). On the contrary, dsGCN5‐treated 13th instar normally molted to the final 14th instar, like the dsGFP controls (Figure 6B). In adult *P. americana*, *GCN5* RNAi led to suppressed suc‐H3^K23^ (Figure 6C; Figure S13B). *GCN5* knockdown and consequent suppression of suc‐H3^K23^ resulted in significantly reduced *Vg* expression (Figure 6D), along with arrested ovarian development and blocked oocyte maturation (Figure 6E). Furthermore, the capacity of JH to induce *Vg* expression was eliminated by *GCN5* RNAi (Figure 6F).

In holometabolous *H. armigera*, suc‐H3^K14^ was suppressed by *P300* knockdown too (Figure 6G; Figure S13C). Loss of *P300* function in the penultimate fifth instar larvae resulted in precocious pupation, which was reminiscent of phenotypes caused by precocene treatment (Figure 6H). In adult *H. armigera*, suc‐H3^K23^ was inhibited by knocking down *GCN5* (Figure 6I; Figure S13D). dsGCN5‐treated adult *H. armigera*, which lacked suc‐H3^K23^, showed a significant decline of *Vg* expression (Figure 6J) as well as blocked ovarian development and retarded oocyte maturation (Figure 6K). Depletion of *GCN5* prevented JH induction of *Vg* expression (Figure 6L).

A previous study has reported that H3^K14A^ mutants of *D. melanogaster* displayed embryonic lethality, whereas H3^K23A^ mutant flies were viable but with significantly reduced fertility [20]. As JH III bisepoxide (JHB3) is the primary form of JH in *D. melanogaster* [51], we further examined the effect of JHB3 on suc‐H3^K14^ and suc‐H3^K23^. Application of JHB3 induced suc‐H3^K14^ in the larvae and suc‐H3^K23^ in the adults (Figure S14A). H3^K23A^ mutation caused significantly decreased expression of *yolk proteins* (Figure S14B), along with arrested ovarian development (Figure S14C). Compared to the wildtypes (W^1118^), H3^K23A^ mutants laid substantially fewer eggs (Figure S14D). Collectively, the observations imply the evolutionary conservation of suc‐H3^K14^ and suc‐H3^K23^ in diverse insect species.

### 2.5 Suc‐H3^K14^ and Suc‐H3^K23^ are Stage‐Specifically Regulated by GPCR‐Mediated Signaling Cascades

**FIGURE 7:**
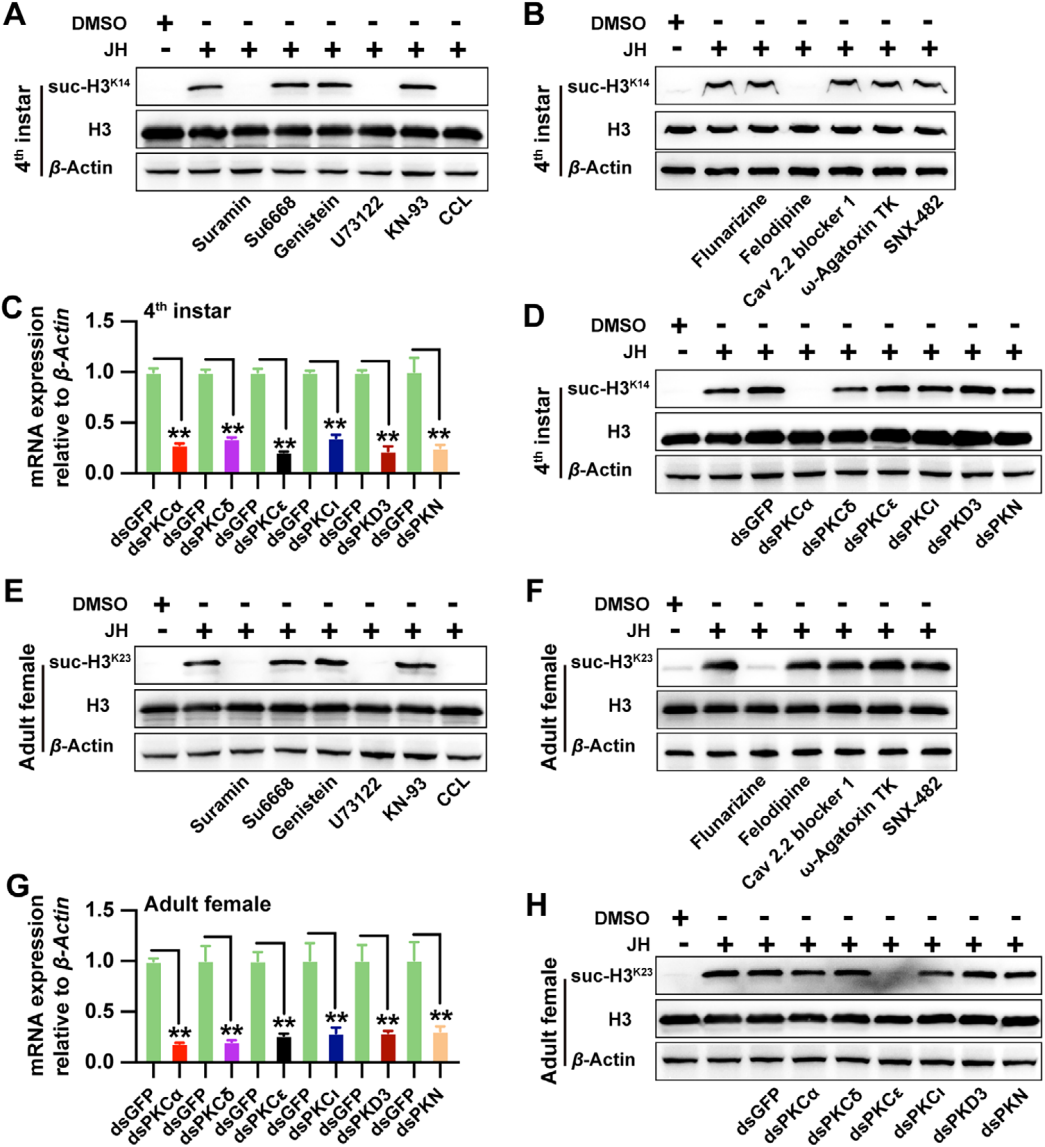
Regulation of suc‐H3^K14^ and suc‐H3^K23^ by the GPCR‐RTK‐PLC‐PKC cascade. (A) Effect of GPCR (Suramin), RTK (SU6668/genistein), PLC (U73122), CaMKII (KN‐93), and PKC (CCL) inhibitors on JH‐induced suc‐H3^K14^ in the fourth instar nymphs. (B) Effect of Ca^2+^ channel blockers (felodipine, L‐type; flunarizine, T‐type; cav2.2, N‐type; ω‐Agatoxin TK, P/Q‐type; and SNX‐482, R‐type) on suc‐H3^K14^. (C) Knockdown efficiency of *PKC* isoforms in the fat body of fourth instar nymphs. ^**^, *p* < 0.01; *n* = 3. (D) Effect of *PKC* isoform knockdown on suc‐H3^K14^. (E, F) Effect of GPCR, RTK, PLC, CaMKII, and PKC inhibitors (*E*) and Ca^2+^ channel blockers (*F*) on JH‐induced suc‐H3^K23^ in the adult females. (G) Knockdown efficiency of *PKC* isoforms in the adult females. ^**^, *p* < 0.01; *n* = 3). (H) Effect of *PKC* isoform knockdown on suc‐H3^K23^ in the adult females.

We next explored the molecules involved in P300‐catalyzed suc‐H3^K14^ and GCN5‐mediated suc‐H3^K23^. Previous studies have demonstrated that JH acts via the GPCR‐PLC‐PKC cascade to regulate insect metamorphosis and reproduction [15, 17]. Since suc‐H3^K14^ and suc‐H3^K23^ are JH‐responsive (Figure 2C,D), we next investigated the involvement of signaling molecules, including GPCR, PLC, and PKC. As shown in Figure 7A,B, injection of suramin (GPCR inhibitor), U73122 (PLC inhibitor), felodipine (blocker of L‐type Ca^2+^ channel, LTCC), and chelerythrine chloride (CCL, PKC inhibitor) abolished JH‐induced suc‐H3^K14^ (Figure 7A,B). Nevertheless, application of SU6668 (RTK inhibitor), genistein (RTK inhibitor), KN‐93 (CaMKII inhibitor), flunarizine (blocker of T‐type Ca^2+^ channel, TTCC), cav2.2 blocker 1 (N‐type Ca^2+^ channel blocker), ω‐Agatoxin TK (P/Q‐type Ca^2+^ channel blocker), or SNX‐482 (R‐type Ca^2+^ channel blocker) had no obvious effect (Figure 7A,B). The data suggest the involvement of GPCR, PLC, LTCC, and PKC in JH‐induced suc‐H3^K14^ in the nymphs. To define the specific PKC isoforms, we performed RNAi‐mediated knockdown of *PKCα*, *PKCε*, *PKCδ*, *PKCι*, *PKD3*, and *PKN* (Figure 7C). Depletion of *PKCα* but neither of the other five isoforms eliminated JH‐induced suc‐H3^K14^ in the fourth instar nymphs (Figure 7D).

As shown in Figure 7E,F, the inhibitors suramin, U73122, and CCL abolished JH‐induced suc‐H3^K23^ in the adults. However, flunarizine but not felodipine treatment led to blocked induction of suc‐H3^K23^ (Figure 7F), and only *PKCε* knockdown eliminated JH‐induced suc‐H3^K23^ in the adults (Figure 7G,H). These observations suggest the involvement of GPCR, PLC, TTCC, and *PKCε* in suc‐H3^K23^ catalyzation. As P300 and GCN5 promoted suc‐H3^K14^ and suc‐H3^K23^ (Figure 3A–D), and these two succinyltransferases are activated via phosphorylation [52, 53], we next investigated PKC‐mediated phosphorylation using immunoprecipitation and western blotting with phospho‐(Ser) PKC substrate antibody. As shown in Figure S15A,B, JH‐stimulated and PKC‐mediated phosphorylation of P300 and GCN5 was observed. Interestingly, PKC‐mediated phosphorylation of P300 and GCN5 was modulated by JH at different levels (Figure S15A,B). Notably, *PKCα* knockdown abolished JH‐induced P300 phosphorylation in the nymphs (Figure S15C,c^1^), and *PKCε* knockdown eliminated JH‐induced GCN5 phosphorylation in the adults (Figure S15D,d^1^). The results indicate that PKCα and PKCε are required for JH‐stimulated phosphorylation of P300 and GCN5, respectively. Developmental profiling showed that *PKCα* transcript levels were remarkably higher in the fourth instar nymphs compared to adults (Figure S16A). In contrast, *PKCε* expression was significantly higher in adults than in fourth instar nymphs (Figure S16B). Such patterns were not observed with *PKCδ*, *PKCι*, *PKD3* or *PKN* (Figure S16C–F). These data suggest that the selection of PKC isoforms may correlate with their stage‐specific abundance. Collectively, the above results suggest that suc‐H3^K14^ is catalyzed by the GPCR‐PLC‐LTCC‐PKCα‐P300 cascade during the nymphal stage, whereas suc‐H3^K23^ is promoted by the GPCR‐PLC‐TTCC‐PKCε axis in adulthood.

## 3 Discussion

Histone lysine succinylation has been established as a metabolism‐linked epigenetic modification, with its abundance tightly coupled to the level of its primary donor, succinyl‐CoA [54, 55]. Succinyl‐CoA is mainly derived from the tricarboxylic acid cycle and amino acid metabolism, particularly the catabolism of leucine, isoleucine, and valine [56]. Knockout of *succinyl‐CoA synthetase/ligase alpha subunit* (*Scsα*) led to developmental delays, locomotor activity defects, and reduced survival under starvation in *D. melanogaster* [55], indicating the physiological importance of succinyl‐CoA in insects. We observed that succinyl‐CoA was among the top three CoAs, and the pathway of leucine, isoleucine, and valine metabolism was at the top of metabolic pathways in the fat body. Furthermore, the abundance of lysine succinylation was significantly higher than acetylation, crotonylation, 2‐hydroxy‐isobutyrylation, and malonylation. It is noteworthy that lysine succinylation was more abundant than lysine acetylation, though the levels of succinyl‐CoA were lower than those of acetyl‐CoA. Compared with acetylation, succinylation alters more dramatically with a greater change in charge and addition of a larger structural moiety, which may contribute to more abundant succinylation [2, 4, 57]. Insect fat body, analogous to vertebrate liver and adipose tissue, is the central hub for nutritional storage, energy metabolism, and protein synthesis [21]. Our above observations are in accordance with the previous findings showing that succinyl‐CoA and lysine succinylation are abundant in vertebrate liver and brown adipose tissue [57, 58].

By using succinyl‐proteome and LC‐MS/MS analysis, we identified 4 succinylation sites of H3K residues, including suc‐H3^K14^, suc‐H3^K23^, suc‐H3^K79^, and suc‐H3^K122^ in the fat body of both nymphs and adults. Notably, suc‐H3^K14^ was more abundant in the penultimate fourth instar nymphs, whereas suc‐H3^K23^ was at high levels in the adult females at the early vitellogenic stage, suggesting the potential involvement of suc‐H3^K14^ in metamorphosis and suc‐H3^K23^ in oogenesis. Considering that the JH titer was much lower in the nymphal stage than in adulthood, we hypothesized that suc‐H3^K14^ and suc‐H3^K23^ were differentially regulated in response to JH. Indeed, suc‐H3^K14^ was induced by a lower level of JH in nymphs, whereas suc‐H3^K23^ was stimulated by a higher titer of JH in adults. Previously, proteomic analyses have identified succinylation sites in other insects, including *B. mori* and *Solenopsis Invicta*, but the role of succinylated proteins was not explored [59, 60]. A recent study demonstrated that the molting hormone 20‐hydroxyecdysone stimulates succinylation of insulin‐degrading enzyme (IDE) at K^179^ to drive tissue remodeling during *H. armigera* metamorphosis [61]. Our present study extends the view of protein succinylation in the regulation of insect metamorphosis and reproduction by identifying H3 lysine succinylation as the key mediator.

Lysine succinylation can be achieved enzymatically by P300, GCN5, HAT1, and CPT1A [9, 11, 27]. In addition to succinylation, GCN5, P300, HAT1, and CPT1A are capable of catalyzing acetylation, crotonylation, 2‐hydroxy‐isobutyrylation, and malonylation of lysine residues in vertebrates [62, 63, 64, 65]. We demonstrated that suc‐H3^K14^ was catalyzed by P300 in nymphs, whereas suc‐H3^K23^ was mediated by GCN5 in adults. Notably, *P300* knockdown and *GCN5* depletion had no obvious effect on acetylation, crotonylation, 2‐hydroxy‐isobutyrylation, or malonylation of H3^K14^ or H3^K23^. The results provide a clear indication that P300 and GCN5 promoted suc‐H3^K14^ and suc‐H3^K23^ in the nymphs and adults, respectively. While *P300* knockdown resulted in precocious metamorphosis, *GCN5* depletion led to significantly reduced *Vg* expression and blocked egg development. It is conceivable that suc‐H3^K14^ plays a pivotal role in JH‐repressed larval metamorphosis, and suc‐H3^K23^ is indispensable for JH‐stimulated adult female reproduction. Nevertheless, the role of other acylations at H3^K14^ and H3^K23^ cannot be excluded since acetylation, crotonylation, 2‐hydroxy‐isobutyrylation, and malonylation were present at these two residues. However, it is noteworthy that P300 and GCN5 primarily induced suc‐H3^K14^ and suc‐H3^K23^, respectively. Moreover, lysine succinylation has a more profound regulatory role than other lysine acylations because of the charge shift from positive to negative and the addition of a larger structural moiety from succinylation [2, 59, 66]. By using CRISPR/Cas9, we further documented that the H3^K14A^ mutation caused embryonic lethality, and the H3^K23A^ mutation abolished JH‐induced *Vg* expression, blocked egg development, and remarkably reduced fecundity. These observations agree with a previous report on *Drosophila*, which demonstrated that H3^K14A^ mutants were embryonically lethal, and H3^K23A^ mutants had significantly declined fertility [20]. It should be pointed out that H3^K14^ and H3^K23^ are also known sites for other PTMs, notably acetylation. We observed that JH stimulated succinylation at H3^K14^ and H3^K23^, but had no significant effect on acetylation, crotonylation, 2‐hydroxy‐isobutyrylation or malonylation at these two residues. Knocking down *P300* and *GCN5* reduced JH‐induced succinylation at H3^K14^ and H3^K23^, respectively, without significant impact on other PTMs. Moreover, precocious metamorphosis caused by *P300* knockdown phenocopied the JH‐deprived condition, and JH‐induced *Vg* expression was blocked by *GCN5* knockdown. Altogether, the data suggest that succinylation at H3^K14^ and H3^K23^ plays a predominant role in the observed phenotypes. Nevertheless, we cannot exclude the involvement of other PTMs.

Cut&Tag‐seq and ChIP‐qPCR analyses provide evidence that suc‐H3^K14^ and suc‐H3^K23^ mediate JH function in metamorphosis and reproduction by targeting distinct gene networks. In the nymphs, suc‐H3^K14^ was enriched in the promoters of genes regulating metamorphosis, cuticle and chitin remodeling, including *E78C*, *HR38*, *BR*, *NCP14.9*, *Abd‐8*, *CP18.7‐L*, *CP*, and *Obst‐E‐like* [32, 33, 35, 38]. In adults, suc‐H3^K23^ targeted the genes regulating vitellogenesis, including *Vg*, *LRP1*, *LRP2*, *ApoLp*, *LpR‐S*, and *OVGP* [14, 45, 46, 49]. Thus, our findings collectively revealed the crucial role of suc‐H3^K14^ in metamorphosis and suc‐H3^K23^ in reproductive capacity. Importantly, P300‐mediated suc‐H3^K14^ and GCN5‐promoted suc‐H3^K23^ were observed in both hemimetabolous and holometabolous insect species, including the orthopteran *L. migratoria*, the blattarian *P. americana*, the lepidopteran *H. armigera*, and the dipteran *D. melanogaster*. These observations demonstrate a remarkable conservation of both the function and the regulation of suc‐H3^K14^ and suc‐H3^K23^ across more than 300 million years of insect evolution, highlighting the significance of H3 lysine succinylation in modulating insect metamorphosis and reproduction.

It is of interest to note that GPCR, PLC, and PKC are involved in P300‐triggered suc‐H3^K14^ and GCN5‐promoted suc‐H3^K23^. The GPCR‐PLC‐PKC cascade has been previously reported to convey JH signaling in repressing precocious metamorphosis and stimulating reproduction [15, 17, 67]. For instance, the GPCR‐PLC‐PKC cascade transduces JH signaling to trigger BR phosphorylation, thus inhibiting precocious larval metamorphosis of *H. armigera* [15]. In adult *L. migratoria*, JH activates atypical PKC (aPKC) via GPCR signaling to regulate intercellular adhesion between follicle epithelial cells and promote patency formation [68]. JH also acts through the GPCR‐PLC pathway to activate PKCι, which subsequently phosphorylates and activates the vitellogenin receptor (VgR) to facilitate Vg endocytosis into oocytes [17]. In the *A. aegypti*, JH treatment activates PKC via PLC, and multiple PKC isoforms are involved in JH action during post‐emergence development of adult females [69]. Notably, while PKCα initiated P300 phosphorylation and activation for suc‐H3^K14^ in the nymphs, PKCε promoted GCN5 phosphorylation and activation for suc‐H3^K23^ in the adults. *PKCα* was expressed in remarkably higher levels in the nymphs compared to adults, whereas *PKCε* expression was significantly higher in the adults than in the fourth instar nymphs. It seems likely that stage‐specific expression patterns of *PKCα* and *PKCε* contribute to stage‐specific catalyzation of suc‐H3^K14^ and suc‐H3^K23^. PKCα belongs to the conventional PKC subfamily sensitive to 1,2‐diacylglycerol (DAG) and calcium, whereas PKCε is a member of the novel PKC subfamily that is DAG‐sensitive but calcium‐insensitive [70, 71, 72]. We observed that the L‐type Ca^2+^ channel was involved in JH‐induced suc‐H3^K14^, and the T‐type Ca^2+^ channel participated in JH‐induced suc‐H3^K23^, implying the stage‐specific variation of voltage‐gated calcium channels in oscillatory calcium influx. L‐type Ca^2+^ channel is characterized by long opening duration and high voltage sensitivity, whereas T‐type Ca^2+^ channel is transient and at low‐voltage depolarization [73, 74, 75]. It also seems likely that different PKC isoforms and Ca^2^^+^ channels contribute to stage‐specific JH responsiveness and succinyltransferase activity for suc‐H3^K14^ and suc‐H3^K23^.

**FIGURE 8:**
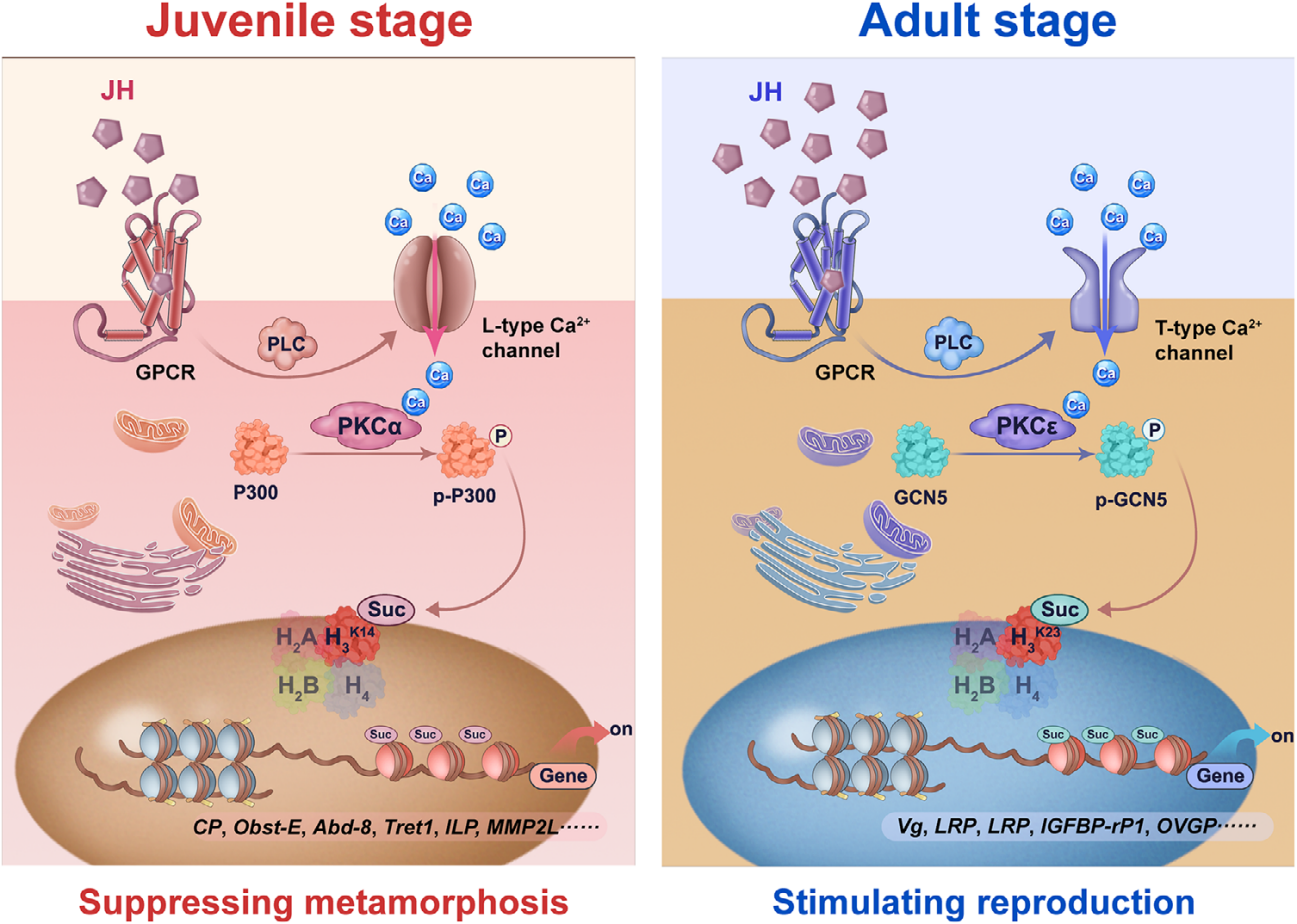
Schematic illustration of suc‐H3^K14^ and suc‐H3^K23^ in JH‐regulated metamorphosis and reproduction. In the juvenile stage, a low level of JH activates the GPCR‐PLC‐LTCC‐PKCα‐P300 pathway to catalyze suc‐H3^K14^, thereby suppressing precocious metamorphosis. In adulthood, high doses of JH act via the GPCR‐PLC‐TTCC‐PKCε‐GCN5 cascade to promote suc‐H3^K23^, hence stimulating reproductive capacity. This stage‐specific mechanism is evolutionarily conserved across divergent insect orders.

In synopsis with our findings, we propose a conceptual model for suc‐H3^K14^ and suc‐H3^K23^ in insect metamorphosis and reproduction (Figure 8). During the nymphal stage, a low level of JH activates the GPCR‐PLC‐LTCC‐PKCα pathway to trigger P300 phosphorylation and activation, thereby catalyzing suc‐H3^K14^ and repressing precocious metamorphosis. In adulthood, high doses of JH act via the GPCR‐PLC‐TTCC‐PKCε cascade to promote GCN5 phosphorylation and activation, subsequently promoting suc‐H3^K23^ and oogenesis. These stage‐specific succinylation events modulate chromatin‐based processes and gene transcription to exert the dual functions of JH in suppressing precocious larval metamorphosis and stimulating adult reproduction.

## 4 Materials and Methods

### 4.1 Animals

The gregarious phase of *L. migratoria* was maintained with wheat bran plus fresh wheat seedlings at 30 ± 2°C and a photoperiod of 14L:10D. *H. armigera* was reared with an artificial diet at 27°C and 14L:10D. *D. melanogaster* strains were fed on a standard cornmeal‐yeast‐agar medium at 25°C and 12L:12D. *P. americana* colony was kept with commercial rat food at 28 ± 1°C and 12L:12D.

Precocene III (Sigma‐Aldrich) was intra‐abdominally injected within 6 h post larval molting or adult emergence at 25 µg per *L. migratoria* nymph, 500 µg per *L. migratoria* adult, 500 µg per *P. americana* nymph, and 50 µg per *H. armigera* larva. JH III (Echelon Biosciences) was injected at 2 µg per *L. migratoria* nymph, 10 µg per *L. migratoria* adult, 10 µg per *P. americana* adult, and 8 µg per *H. armigera* adult. For *D. melanogaster*, JH III bisepoxide (Toronto Research Chemicals) was administered at the doses of 0.1 µg/larva and 0.5/adult.

In pharmacological experiments, the early fourth instar nymphs and previtellogenic adults of *L. migratoria* were injected with suramin (KKL MED; 30 µg, 150 µg), SU6668 (Selleck; 0.6 µg, 3 µg), genistein (MCE; 1.2 µg, 6 µg), U73122 (MCE; 2 µg, 10 µg), KN‐93 (MCE; 0.5 µg, 10 µg), CCL (Selleck; 1 µg, 5 µg), flunarizine (Selleck; 10 µg, 50 µg), Felodipine (TOPSCIENCE; 1 µg, 5 µg), Cav2.2 blocker 1 (TOPSCIENCE; 2 µg, 10 µg), ω‐Agatoxin TK (MCE; 0.25 µg,1 µg) and SNX‐482 (MCE; 0.25 µg, 1 µg), followed by JH or DMSO treatment for 30 min.

### 4.2 RNA‐seq and ssGSEA

RNA‐seq was performed by BGI. Briefly, total RNAs were isolated from the fat body of fourth instar nymphal and adult *L. migratoria*, and cDNA libraries were constructed in single‐read mode using the DNBSEQ platform (BGI). Single‐sample gene set enrichment analysis (ssGSEA) was conducted by Bioyigene. Differentially expressed genes were determined at the criteria of fold change > 2 and *p* < 0.05 using the DESeq2 package. Clean reads were aligned to the reference genome of *L. migratoria* (GCA_026315105.1). Gene ontology (GO) enrichment and KEGG terms were analyzed by Bioyigene. Digital illustrations were generated using Oebiotech's online platform (https://cloud.oebiotech.cn/task/).

### 4.3 Acylated CoA Analysis

The collected fat bodies were ground on ice and mixed with 80% methanol, followed by fortifying with dichlorophenylalanine. LC‐MS/MS was performed by Sensichip using a Waters UPLC system coupled to a Thermo Q Exactive mass spectrometer. Chromatographic separation was conducted on an ACQUITY UPLC HSS T3 column. The mobile phase consisted of solvent A (0.05% formic acid in water) and solvent B (acetonitrile) at a flow rate of 0.3 mL/min, starting with 5% B (0–0.1 min), increasing to 95% B (0.1–12 min), holding at 95% B (12–13.5 min), and then decreasing to 5% B (13.5–13.6 min) and holding at 5% B (13.6–16 min). Mass spectrometry was conducted in electrospray ionization (ESI) mode with the following parameters: heater temperature of 300°C, sheath gas flow rate 45 arbitrary units (arb), auxiliary gas flow rate 15 arb, capillary temperature of 350°C, S‐Lens RF level was set to 30% for positive and 60% for negative ion mode. Full‐scan MS data were acquired over a mass range of m/z 70 to 1050, followed by data‐dependent MS2 scanning (TopN = 10) using high‐energy collision dissociation (HCD).

### 4.4 Capillary‐Based Western Blotting

Capillary‐based western blotting was performed using the ProteinSimple Jess system (Bio‐Techne) according to the manufacturer's manual. Briefly, protein extracts from the fat bodies were mixed with the ProteinSimple Master Mix at a ratio of 0.1:1, and heated at 95°C for 5 min. The samples were then processed with the Ladder, Antibody Diluent II, primary antibodies, HRP‐conjugated secondary antibodies, Luminol‐Peroxide Mix, Wash Buffer, and Separation and Stacking Matrices. Each sample was analyzed with three technical replicates (three capillaries). The capillary electrophoresis, protein immobilization to the capillary wall, blocking, antibody incubation, signal detection, and image/data capture were automatically conducted by the ProteinSimple Jess system. Electropherograms, lane view images, and quantitative results were analyzed by equipped Compass for Simple Western software. The antibodies against lysine acetylation, succinylation, crotonylation, 2‐hydroxy‐isobutyrylation, and malonylation were purchased from PTM‐Biolab.

### 4.5 Succinyl‐Proteomic Analysis

Succinyl‐proteome and LC‐MS/MS analysis were performed by PTM‐Biolab. Briefly, proteins were extracted from the fat bodies, followed by trypsin digestion and desalting. The tryptic peptides were dissolved in mobile phase A (0.1% formic acid and 2% acetonitrile) and separated using an Easy‐nLC1000 UHPLC (Bruker Daltonics). The mobile phase B was an aqueous solution containing 0.1% formic acid. The UHPLC gradient program was set as follows: 9%‐30% mobile phase B (0–44 min), 30%–40% mobile phase B (44–52 min, 40%‐90% mobile phase B (52–56 min), and 90% mobile phase B (56–60 min), with a constant flow rate of 450 nL/min. After UHPLC separation, the peptides were injected into a Capillary Ion Source for ionization and then analyzed using timsTOF Pro mass spectrometry. High‐resolution TOF detection was used to analyze the parent ions and secondary fragments. Following first‐order MS acquisition, 10 parallel accumulative serial fragmentation modes were employed to acquire MS2 spectra of the parent ion with charge numbers ranging from 0 to 5. The dynamic exclusion time for serial MS scanning was set at 24 s to avoid the repeated scanning of the parent ion. MS/MS data were processed with the MaxQuant search engine (v.1.6.15.0). The clean data were aligned to the *L. migratoria* reference genome (GCA_026315105.1) for peptide identification and succinylation site mapping.

### 4.6 Western Blot and Immunoprecipitation

Protein extracts from insects and Sf9 cells were isolated using ice‐cold lysis buffer containing 50 mm Tris‐HCl (pH 7.4), 150 mm NaCl, 0.5% NP‐40, 0.5% sodium deoxycholate, 1 mm PMSF, and protease/phosphatase inhibitor cocktails (Roche). Lysates were cleared by centrifugation, fractionated on a 7.5% SDS‐PAGE, and transferred to a PVDF membrane (Millipore). Western blotting was performed using the primary antibodies, corresponding HRP‐conjugated secondary antibodies (ProteinTech), and an ECL Chemiluminescence Detection Kit (Boster). For immunoprecipitation, precleared lysates from Sf9 cells transfected with pIEx‐4‐P300‐RFP‐His and further treated with 0.1 µm JH or pIEx‐4‐GCN5‐RFP‐His further treated with 5 µm JH were incubated with His‐tag antibody overnight at 4°C. The immunocomplexes were captured with protein‐A agarose beads (Beyotime) at 4°C for 4 h and subsequently eluted in Laemmli sample buffer, followed by western blotting. Antiserum against locust *β*‐Actin was raised as previously described [67]. Antibodies against suc‐H3^K14^, 2‐Hyd‐H3^K14^, Cro‐H3^K14^, Ace‐H3^K14^, suc‐H3^K23^, Mal‐H3^K23^, 2‐Hyd‐H3^K23^, Cro‐H3^K23^, Ace‐H3^K23^, suc‐H3^K79^, suc‐H3^K122^, phospho‐(S) PKC substrate, P300, GCN5, and His‐tag were purchased from Abcam, CST, ZSGB‐Bio, PTM‐Biolab, and Proteintech.

### 4.7 Juvenile Hormone Quantification

The procedure was modified from a previously described protocol [26]. Briefly, the hemolymph was mixed with 70% methanol, followed by centrifugation at 4,500 × *g* for 10 min. The supernatant was then analyzed by LC‐MS/MS using a Waters Acquity UPLC system coupled to an AB SCIEX 5500 QTrap tandem mass spectrometer (Sensichip). Chromatographic separation was achieved on an Acquity UPLC BEH C18 column. Mass spectrometric detection was conducted in ESI‐positive mode with multiple reaction monitoring (MRM) to track JH III‐specific parent‐to‐product ion transitions. Data acquisition and processing were performed with the MultiQuant software.

### 4.8 RNA Interference

cDNA templates were amplified by PCR, cloned into the pGEM‐T vector (Tiangen), and confirmed by sequencing. dsRNAs were synthesized using the T7 RiboMAX Express RNAi System (Promega). Newly‐emerged nymphs/larvae or adults were intra‐abdominally injected with dsRNA at the doses of 2 µg/fourth instar nymph and 15 µg/adult of *L. migratoria*, 10 µg/13th instar nymph and 10 µg/adult of *P. americana*, 5 µg/fifth instar larva and 2 µg/adult of *H. armigera*. Knockdown efficiency was calculated on day 3 for *L. migratoria*, day 6 for *P. americana*, and day 2 for *H. armigera*. In JH treatment, JH III was applied at 2 µg/nymph and 10 µg/adult for 1–6 h. Phenotypes were photographed using a Canon EOS550D camera and a Leica M205C stereomicroscope. Primers used for dsRNA synthesis are listed in Table S1.

### 4.9 qRT‐PCR

Total RNA was extracted from the fat bodies using VeZol reagent (Vazyme). cDNA was reverse transcribed using the FastQuant RT Kit with gDNase (Tiangen). qRT‐PCR was performed using the SuperReal PreMix Plus kit with SYBR Green I (Tiangen) on a Light Cycler 96 System (Roche), initiated at 95°C for 2 min, followed by 40 cycles of 95°C for 20 s, 58°C for 20 s, and 68°C for 20 s. Relative gene expression levels were calculated using the 2^−∆∆Ct^ method and normalized to β‐actin. Primers used in qRT‐PCR are listed in Table S1.

### 4.10 CRISPR/Cas9‐Mediated Knockout and Mutation

The H3^K23A^ mutant line of *D. melanogaster* was generated as previously described [20]. For H3^KO^, H3^K14A^, and H3^K23A^ of *L. migratoria*, freshly laid eggs were collected from the egg pods, washed with 70% ethanol, and placed on the injection pad. Single‐guide RNAs (sgRNAs) were synthesized using the Precision gRNA Synthesis Kit (Invitrogen, A29377). Two 99‐nt single‐stranded oligodeoxynucleotide (ssODN) donors with the targeted lysine substituted to alanine were synthesized by Shanghai Sangon Biotech. Cas9 protein (Invitrogen, A36496), sgRNA, and ssODN were then mixed at the final concentrations of 2.5 µm, 11.5 µm, and 50 µm. A 30 nL mixture was injected into the eggs using a TransferMan 4r micromanipulator (Eppendorf) equipped with a glass micropipette under a dissecting microscope. The injected eggs were incubated at 30°C till the nymphs hatched, and the nymphs were reared with wheat bran plus fresh wheat seedlings at 30 × 2°C and 14L:10D photoperiod. For genotyping, the hemolymph was collected and lysed with 50 mm NaOH at 95°C for 30 min, neutralized with 1 m Tris‐HCl (pH 8.0). An aliquot of supernatants was used as a template for PCR to screen target alleles, with positive clones verified by Sanger sequencing. Mutant lines were backcrossed with the wildtypes for at least five generations to eliminate potential off‐target effects. Primers for sgRNA synthesis, ssODN donor, and genotyping are listed in Table S1.

### 4.11 Cut&Tag‐seq and RNA‐Seq Analyses

The library was constructed following the CUT&Tag Library Prep Kit manual (Vazyme). Nucleus extracts were isolated from early fourth instar nymphs (suc‐H3^K14^) and 3‐day‐old adult females (suc‐H3^K23^) of locusts treated with JH III at 2 µg per nymph and 10 µg per adult for 6 h, using the Nuclear Isolation Kit (SHBIO). Genomic DNA was quantified with a Qubit Fluorometer and amplified with Illumina Index Primers (Vazyme). PCR products were purified with DNA Clean Beads (Vazyme) for sequencing. High‐throughput sequencing was conducted by Wancheng Gene using an Illumina NovaSeq platform, and aligning clean reads to the reference genome (GCA_026315105.1). Statistical analyses were carried out by Origine with biological triplicates and at *p* < 0.05. Differential peaks were identified using MACS, and peaks were annotated to genomic regions (promoters at TSS ± 2 kb, introns, exons, and intergenic regions) using HOMER. RPKM was calculated to generate TSS‐proximal binding profiles and heatmaps of signal distribution across gene bodies. For parallel RNA‐seq, total RNA was isolated from the fat bodies of early fourth instar nymphal and 3‐day‐old adult female locusts treated with JH III at 2 µg per nymph and 10 µg per adult for 6 h. cDNA libraries were constructed and sequenced on the DNBSEQ‐T7 platform (BGI). Clean reads were aligned to the *L. migratoria* reference genome (GCA_026315105.1). Differential gene expression was analyzed by integrating CUT&Tag peaks with RNA‐seq data using DESeq2, with the criteria of fold change > 1.5 and *P *< 0.05. GO enrichment and KEGG pathway annotation were analyzed by Bioyigene.

### 4.12 Chromatin Immunoprecipitation (ChIP) and ChIP‐qPCR

ChIP assays were performed using the Chromatin IP Kit (gzcsbio), and under the conditions in parallel with Cut&Tag‐seq and RNA‐seq analyses. Briefly, fat bodies were collected from early fourth instar nymphs and 3‐day‐old adult females of locusts, treated with JH III at 2 µg per nymph and 10 µg per adult for 6 h. Tissues were cross‐linked with 1% formaldehyde for 10 min at room temperature, then quenched by adding glycine at 125 mm. Chromatin was digested with micrococcal nuclease to obtain the fragments of approximately 150–500 bp. Immunoprecipitation was performed using antibodies against suc‐H3^K14^ or suc‐H3^K23^ overnight at 4°C, with Protein A+G incubation for 2 h at 4°C. Chromatin fragments were then eluted, and cross‐links were reversed by adding 200 mM NaCl at 65°C for 2 h, followed by proteinase K treatment for 1 h. DNA was extracted and analyzed by qPCR using the primers specific for the promoter regions of target genes (Table S1). The percentage of chromatin input was calculated with the formula of 100 × 2^−{Ct^ChIP^−[Ct^Input^−log^2^(input dilution factor)]}^. Ct_ChIP_ and Ct_Input_ represent the Ct of qRT‐PCR from antibody precipitates and the Ct of qRT‐PCR before immunoprecipitation, respectively. Input dilution factor is (fraction of input chromatin saved)^−1^.

### 4.13 Data Analysis

Statistical analyses were performed using GraphPad Prism 10 software. Comparisons between two groups were made using Student's *t*‐test, and multiple comparisons were analyzed using one‐way ANOVA followed by Tukey's post hoc test. The heatmap was generated using online Oebiotech (https://cloud.oebiotech.cn/task/). Graphs were prepared using GraphPad Prism 10 and Adobe Illustrator 2021. Data are presented as mean ± standard deviation (SD).

## Author Contributions

Y.J. and S.Z. designed research; Y.J., L.L., L.Y., L.Z., Q.Y., C.Z., Z.W., G.Y., and Q.L. performed research; Y.J. and S.Z. analyzed data; and S.Z. and Y.J. wrote the paper.

## Conflicts of Interest

The authors declare no conflicts of interest.

## Supporting information




**Supporting File**: advs75624‐sup‐0001‐SuppMat.docx.

## Data Availability

The data that support the findings of this study are available in the supplementary material of this article.
