# Supplementary material for: Stage‐Specific H3K14 and H3K23 Succinylation Orchestrates Insect Metamorphosis and Oogenesis
Source: Adv Sci (Weinh). 2026 May 10;13(43):e75624. doi: 10.1002/advs.75624 (PMC13336120; doi:10.1002/advs.75624)
Supplement: Supplementary file 1 — Supporting File: advs75624‐sup‐0001‐SuppMat.docx. [file ADVS-13-e75624-s001.docx]

**Supplementary to:**

**Stage-specific H3K14 and H3K23 succinylation orchestrates insect metamorphosis and oogenesis**

Yu-Pu Jing^#1^, Lunjie Li^#1,2^, Libin Yang^#1^, Lin-Jie Zhang^1^, Qiang Yan^1^, Ci Zhang^1^, Zanrong Wen^1^, Guangze Yu^1^, Qian Li^1^, Shutang Zhou^*1^

^1^ State Key Laboratory of Cotton Bio-breeding and Integrated Utilization, School of Life Sciences, Henan University, Kaifeng, China

^2^ HLA Laboratory, Henan Red Cross Blood Center, Zhengzhou, Henan, China

^#^Contributed equally.

^*^Correspondence: szhou@henu.edu.cn.


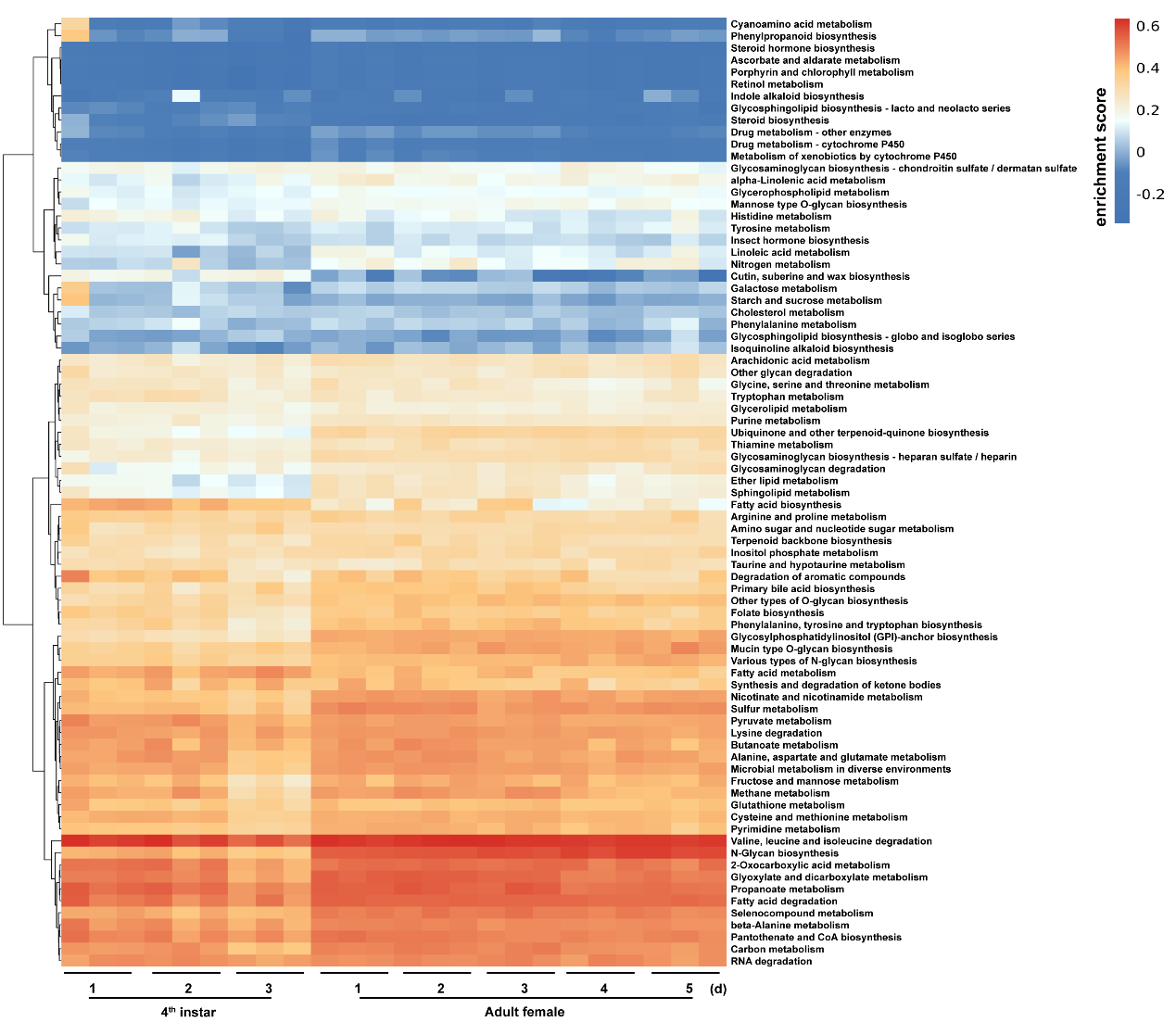


**Figure S1.** **Enriched pathways from single-sample gene set enrichment analysis (ssGSEA).** The metabolic gene sets for ssGSEA were collected from the KEGG terms of RNA-seq on the fat body of penultimate 4^th^ instar nymphs and adult female locusts.


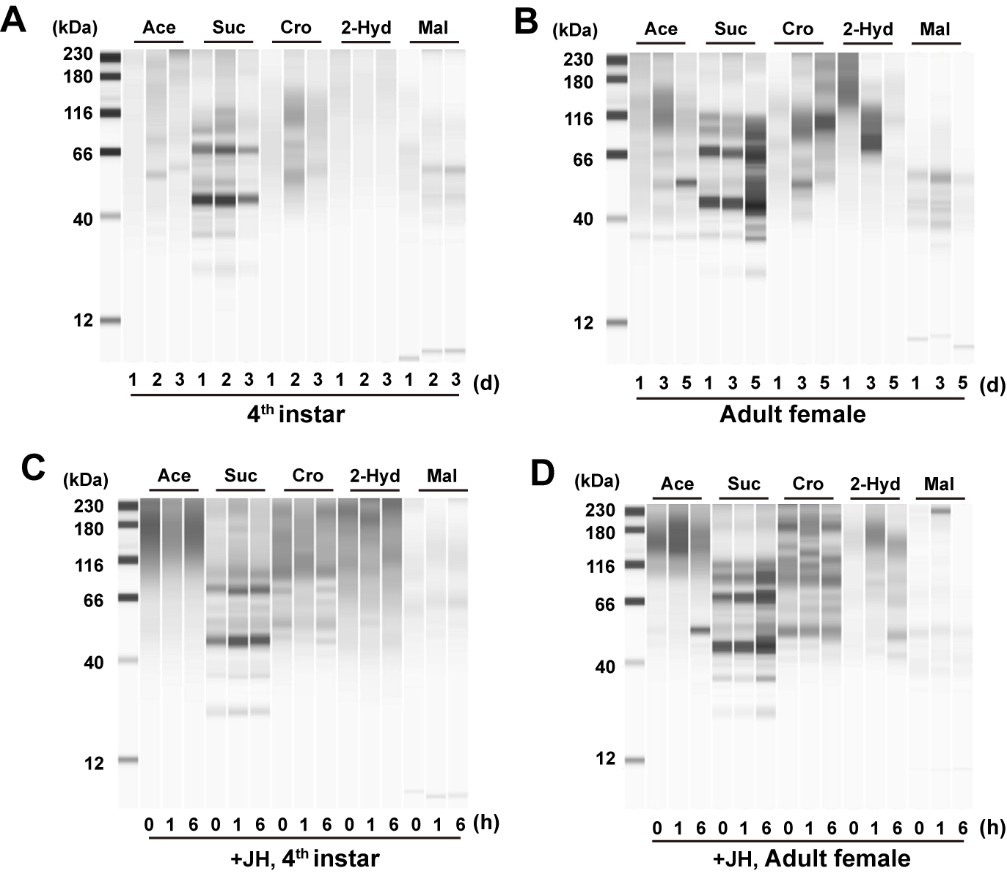


**Figure S2. Simulated images from capillary-based western blotting analysis of lysine acylations in locust fat bodies.** **A.** Lysine acylations in the fat body of penultimate 4^th^ instar nymphs. **B**. Lysine acylations in the fat body of adult females. **C**. Lysine acylations in the fat body of 1-day-old 4^th^ instar nymphs treated with JH at 2 μg for 1 or 6 h. **D**. Lysine acylations in the fat body of 1-day-old adult females treated with JH at 10 μg for 1 or 6 h. Ace, acetylation; Suc, succinylation; Cro, crotonylation; 2-Hyd, 2-hydroxy-isobutyrylation; Mal, malonylation.


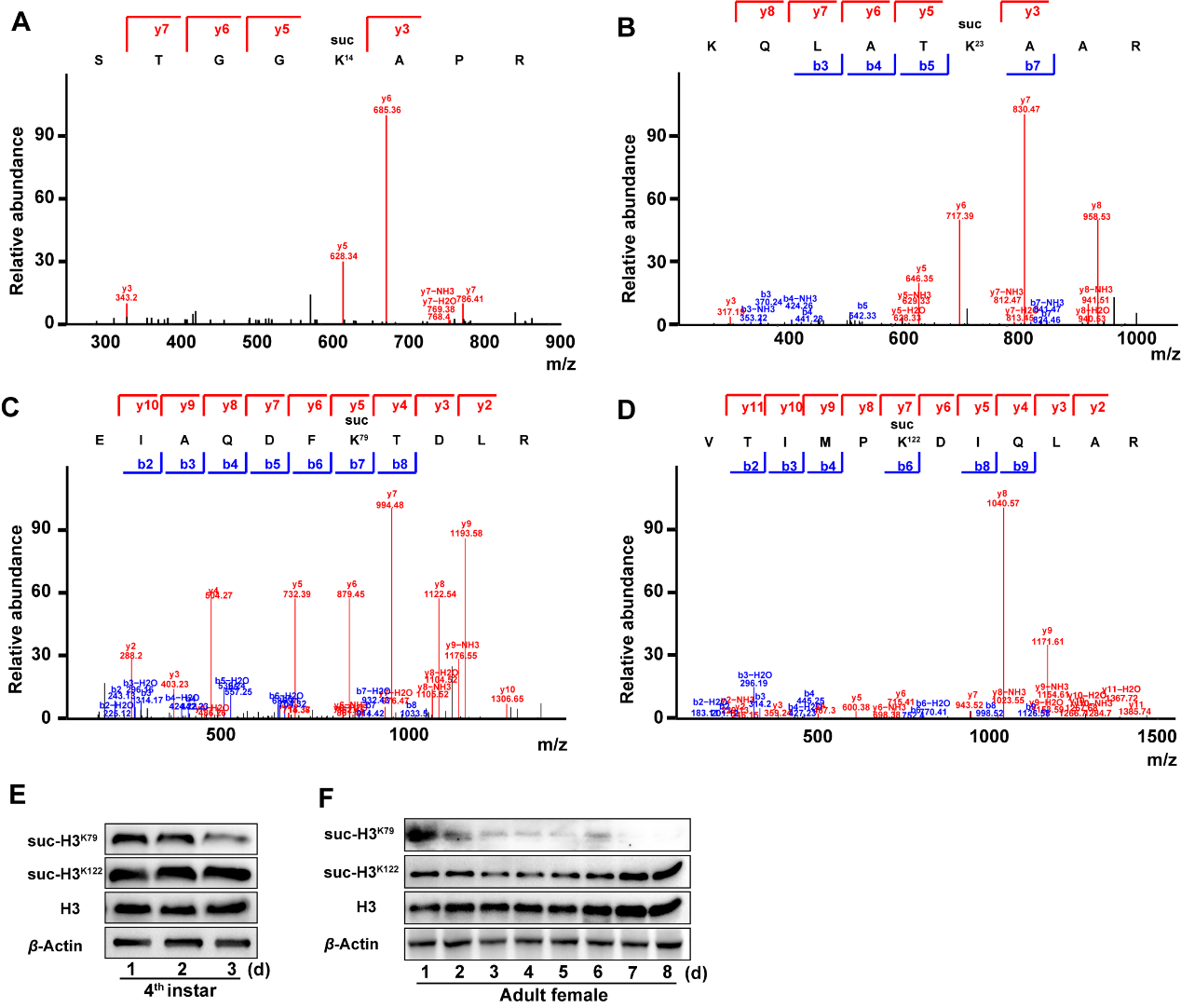


**Figure S3. Identification of H3 succinylation residues in the fat body of 4^th^ instar nymph and adult locusts. A-D.** Identification of succinylation at H3^K14^ (*A*), H3^K23^ (*B*), H3^K79^ (*C*) and H3^K122^ (*D*) by succinyl-proteome and LC-MS/MS analysis of proteins extracted from the fat body of 4^th^ instar nymphs on day 2 and adult females on day 4. **E-F.** Western blot illustrating the developmental abundance of suc-H3^K79^ and suc-H3^K122^ in the fat body of 4^th^ instar nymphs (*E*) and adult females (*F*).


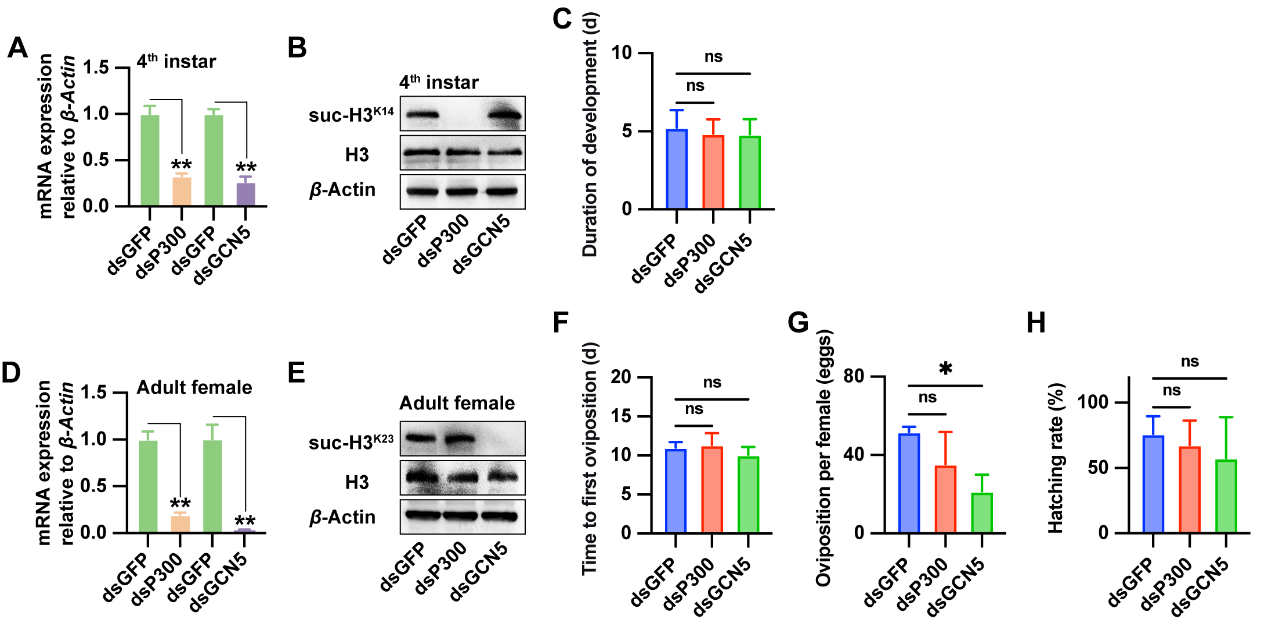


**Figure S4. *P300* and *GCN5* knockdown and effect on suc-H3^K14^ and suc-H3^K23^ in locust fat bodies. A.** Knockdown efficiency of *P300* and *GCN5* in the fat body of penultimate 4^th^ instar nymphal locusts on day 2. **B.** Effect of *P300* and *GCN5* knockdown on suc-H3^K14^ in the fat body of 4^th^ instar nymphal locusts. **C.** Effect of *P300* or *GCN5* knockdown on the developmental duration of nymphs. **D.** Knockdown efficiency of *P300* and *GCN5* in the fat body of adult female locusts on day 6. **E.** Effect of *P300* and *GCN5* knockdown on suc-H3^K23^ in the fat body of adult female locusts. **F.** Effect of *P300* or *GCN5* knockdown on the developmental duration of adults. **G.** Effects *P300* and *GCN5* knockdown on egg number laid per female during the first gonadotrophic cycle. **H.** Effect of *P300* or *GCN5* knockdown on egg hatching rate. dsGFP, the control. ns, no significance; *, *P* < 0.05; **, *P* < 0.01; n = 3.


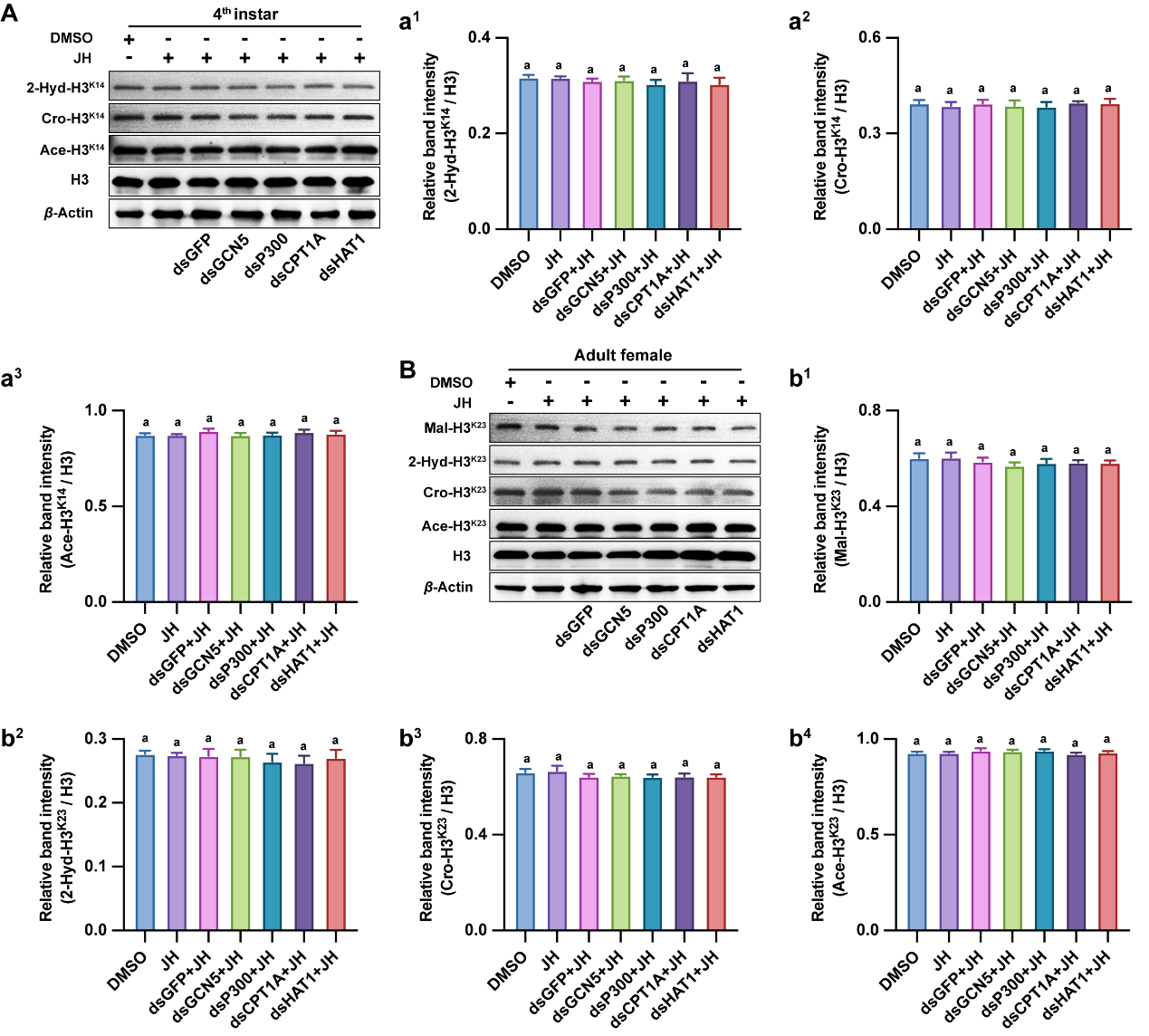


**Figure S5. Effect of *GCN5*, *P300*, *CPT1A* and *HAT1* knockdown on H3^K14^ and H3^K23^ acylations. A**. Western blots showing the abundance of 2-Hyd-H3^K14^, Cro-H3^K14^ and Ace-H3^K14^ in the fat body of 1-day-old 4^th^ instar nymphal locusts subjected to *GCN5*, *P300*, *CPT1A* and *HAT1* RNAi and further treated with 2 μg JH III for 1 h. **a^1^-a^3^.** Quantification of band intensities from three independent experiments of (*A*). **B**. Effect of *GCN5*, *P300*, *CPT1A* and *HAT1* knockdown on Mal-H3^K23^, 2-Hyd-H3^K23^, Cro-H3^K23^ and Ace-H3^K23^ in the fat body of 3-day-old adult females treated with 10 μg JH III for 1 h. **b^1^-b^4^.** Quantification of band intensities from three independent experiments of (*B*). Ace, acetylation; Cro, crotonylation; 2-Hyd, 2-hydroxy-isobutyrylation; Mal, malonylation. DMSO, solvent control. Same letters in a graph indicate no significant difference at *P* < 0.05; n = 3.


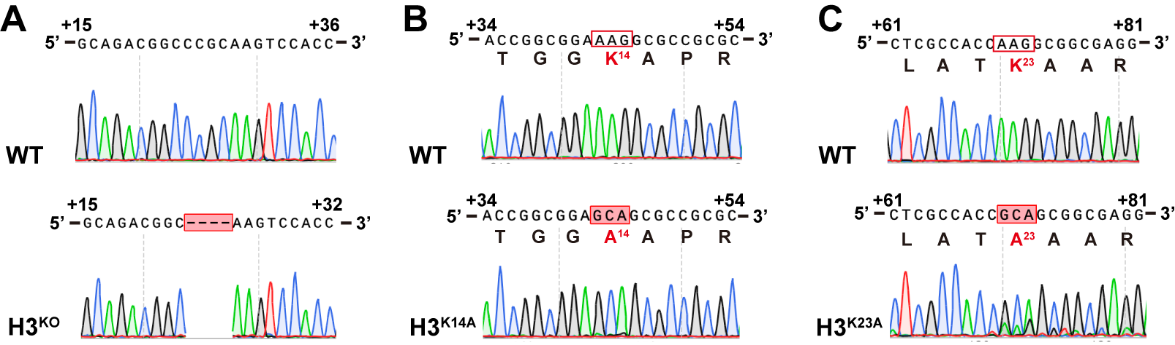


**Supplementary Figure S6. Generation of locust H3^K14^ and H3^K23^ mutations.** DNA sequences showing locust H3 knockout (H3^KO^) site (*A*) as well as mutation sites at H3^K14^ (*B*) and H3^K23^ (*C*). WT, wildtype.


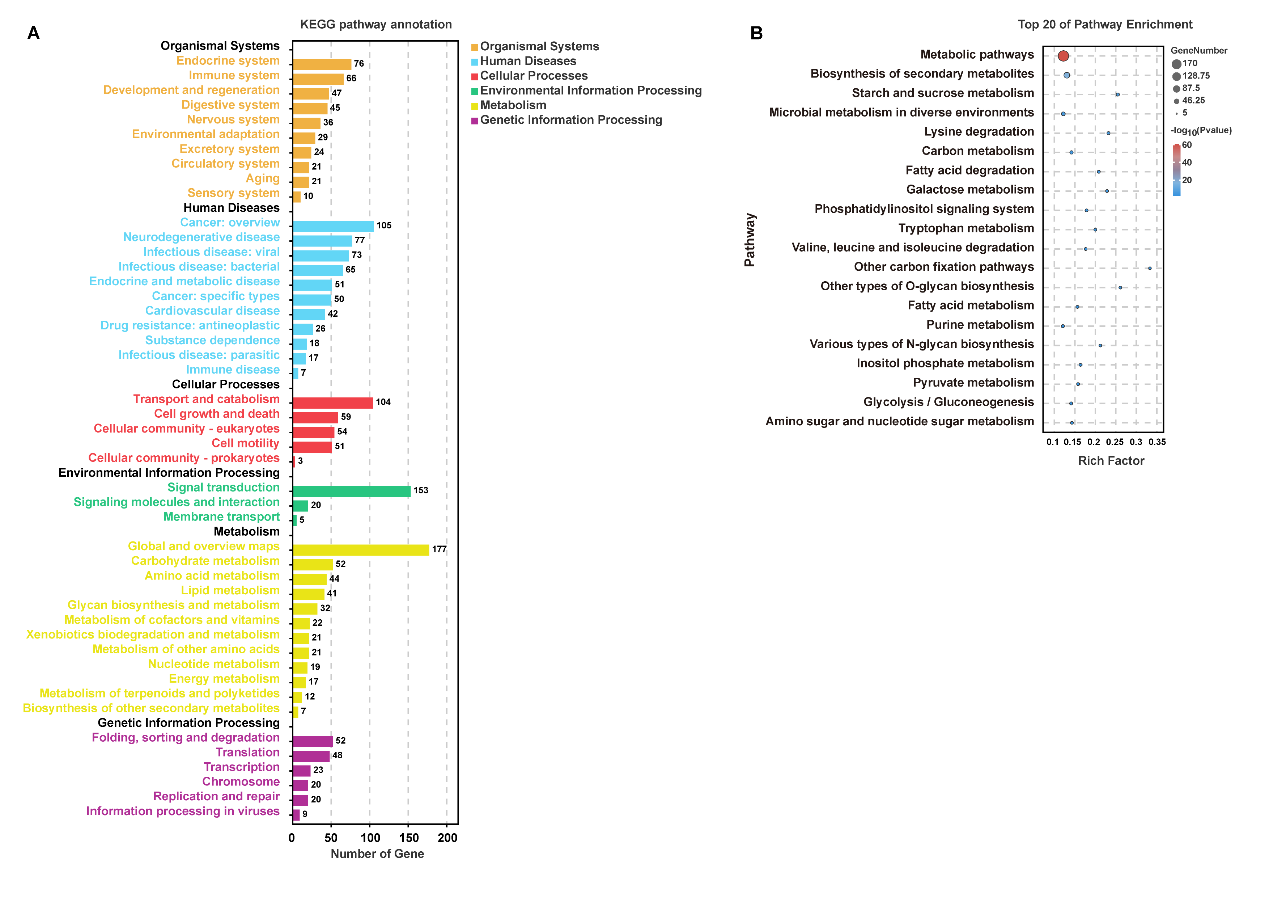


**Figure S7. KEGG pathway enrichment analysis of genes targeted by suc‑H3^K14^. A.** KEGG pathway annotation of differentially expressed genes in the fat body of 4^th^ instar nymphs of locusts from Cut&Tag‑seq and RNA-seq analyses (fold change > 1.5, *P* < 0.05). **B.** Top 20 enriched KEGG pathways among metabolism‑related genes.


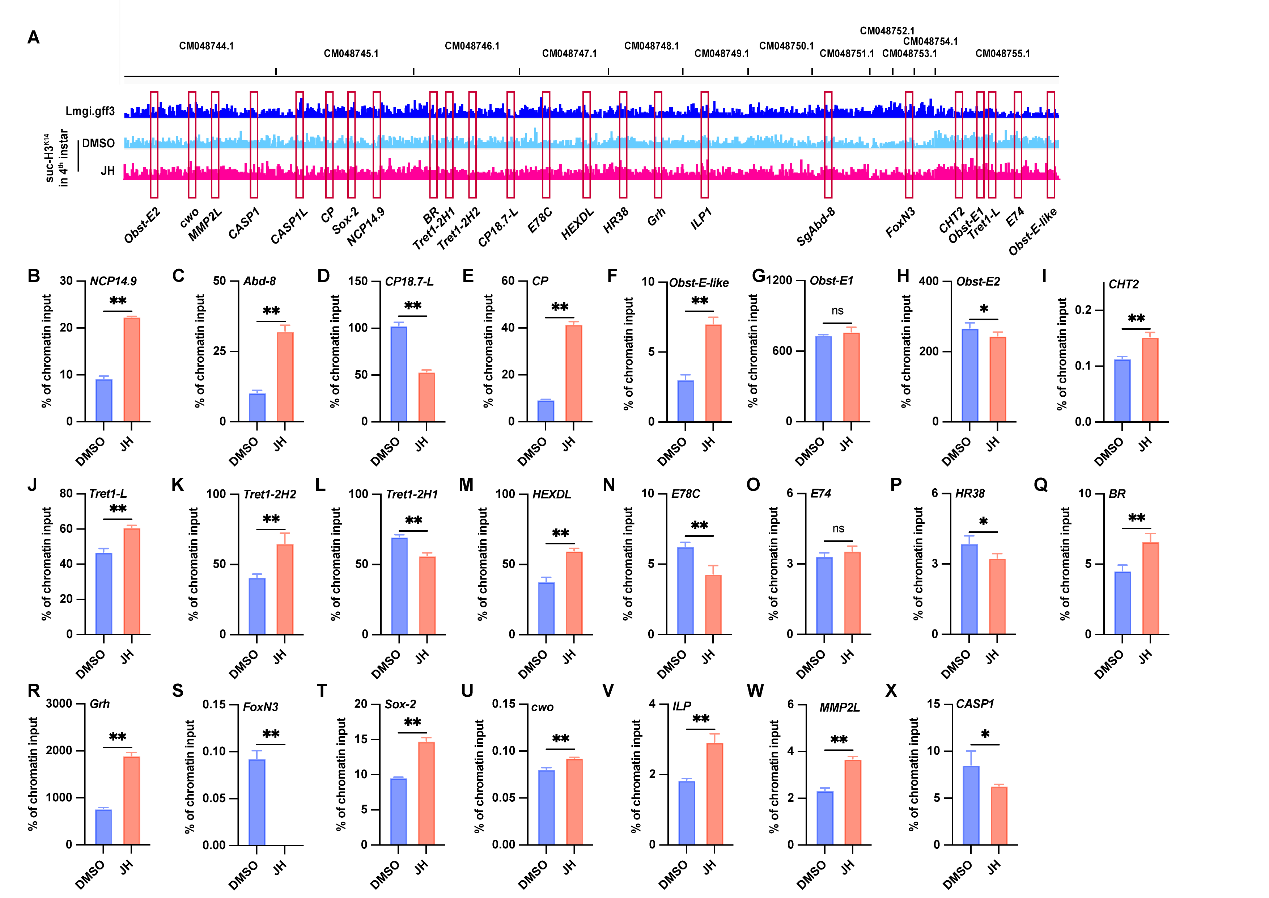


**Figure S8. IGV tracks and ChIP‑qPCR validation of suc‑H3^K14^ enrichment in the promoter regions of JH‑responsive genes in nymphal locusts.** **A.** IGV tracks showing JH‑induced suc‑H3^K14^ enrichment at selected target genes identified by integrative analysis of Cut&Tag‑seq and RNA‑seq on the fat body of 4^th^ instar nymphs treated with JH III vs. DMSO at 2 μg for 6 h. Genomic coordinates and gene annotations are shown at the top. **B-X.** ChIP‑qPCR validation of selected target genes. ChIP was performed using an antibody against suc‑H3^K14^, followed by qPCR analysis. DMSO, solvent control. *, *P* < 0.05; **, *P* < 0.01; ns, not significant. n = 3.


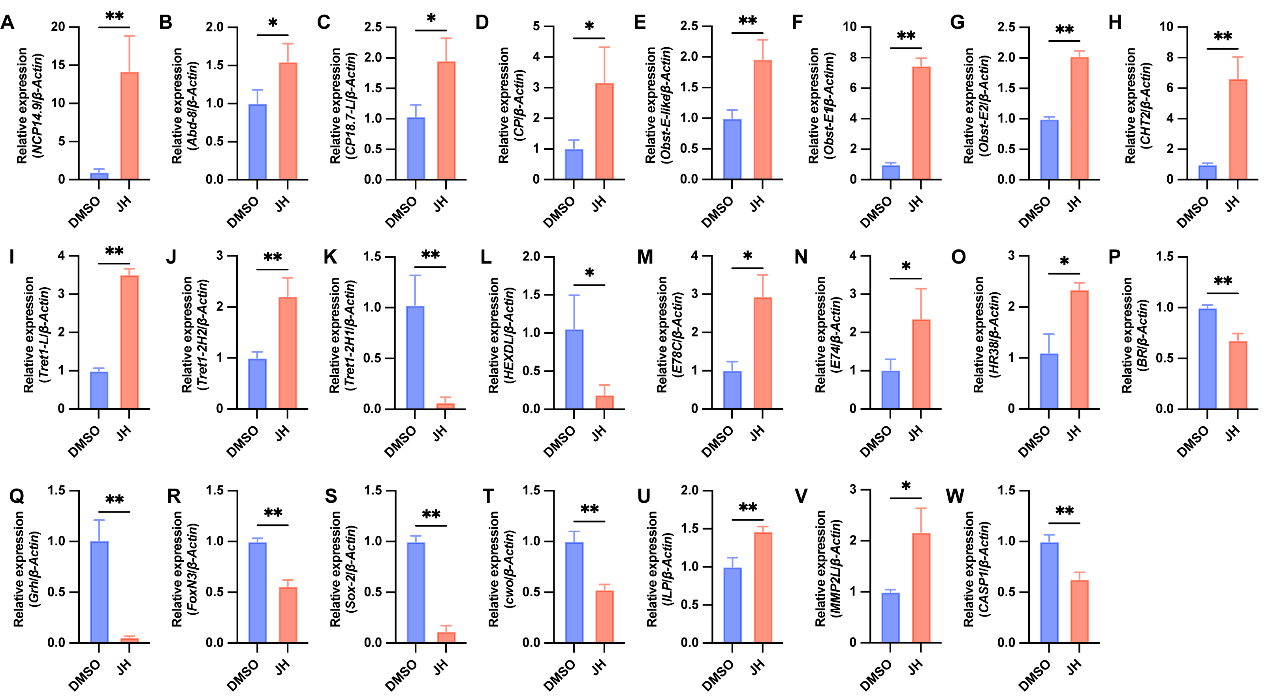


**Figure S9. qRT‑PCR validation of JH‑responsive gene expression in nymphal locusts.** Total RNA was extracted from the fat bodies of 1‑day‑old 4^th^ instar nymphs of locusts treated with JH III at 2 μg for 6 h. Relative expression was normalized to *β*‑*Actin*. DMSO, solvent control. *, *P*< 0.05; **, *P* < 0.01; ns, not significant; n = 3.


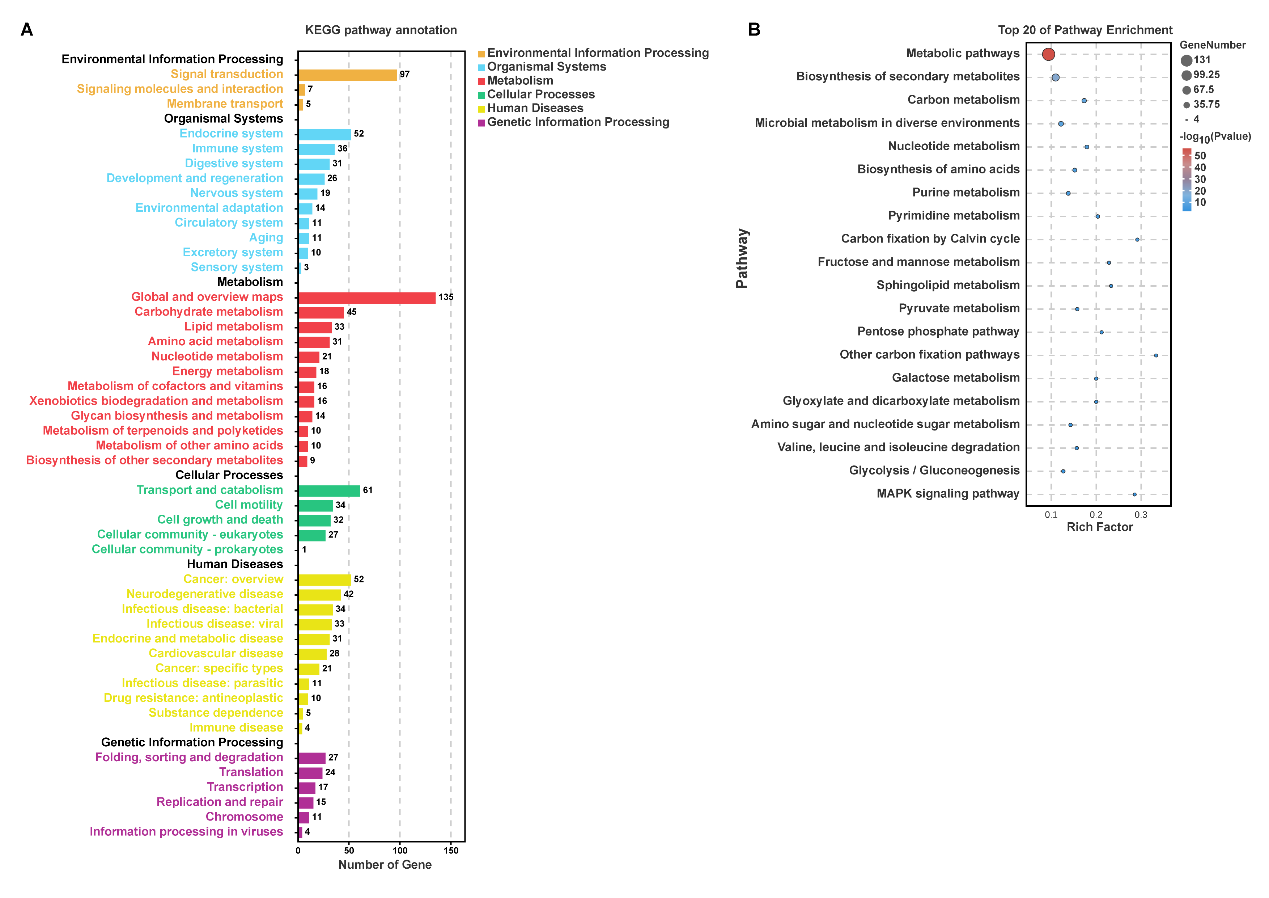


**Figure S10. KEGG pathway enrichment analysis of genes targeted by JH‑induced suc‑H3^K23^. A.** KEGG pathway annotation of differentially expressed genes in the fat body of 3-day-old adult locusts from Cut&Tag‑seq and RNA-seq analyses (fold change > 1.5, *P*< 0.05). **B.** Top 20 enriched KEGG pathways among metabolism‑related genes.


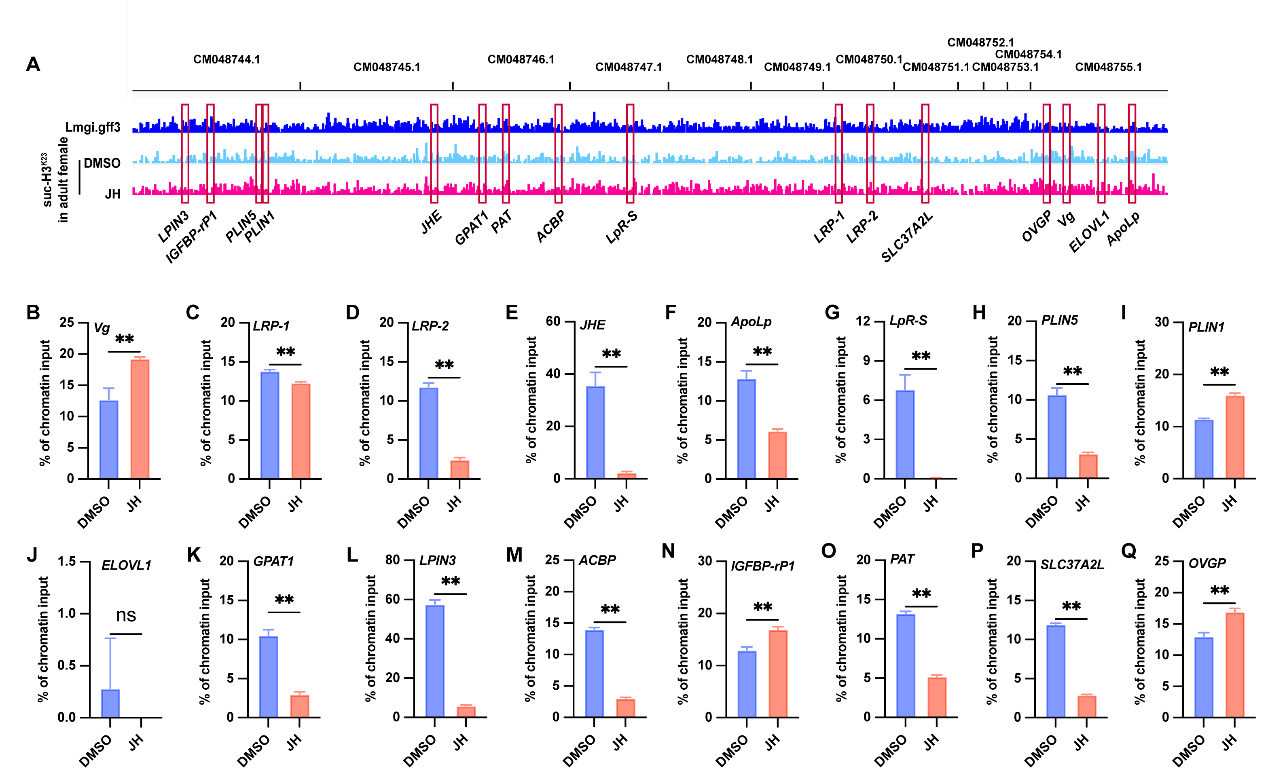


**Figure S11. IGV tracks and ChIP‑qPCR validation of suc‑H3^K23^ enrichment in the promoter regions of JH‑responsive genes in adult locusts. A.** IGV tracks showing JH‑induced suc‑H3^K23^ enrichment at selected target genes identified by integrative analysis of Cut&Tag‑seq and RNA‑seq on the fat body of 3-day-old adult females treated with JH III at 10 μg for 6 h. Genomic coordinates and gene annotations are shown at the top. **B-Q.** ChIP‑qPCR validation of selected target genes. ChIP was performed using an antibody against suc‑H3^K23^, followed by qPCR analysis. DMSO, solvent control. *, *P* < 0.05; **, *P* < 0.01; ns, not significant; n = 3.


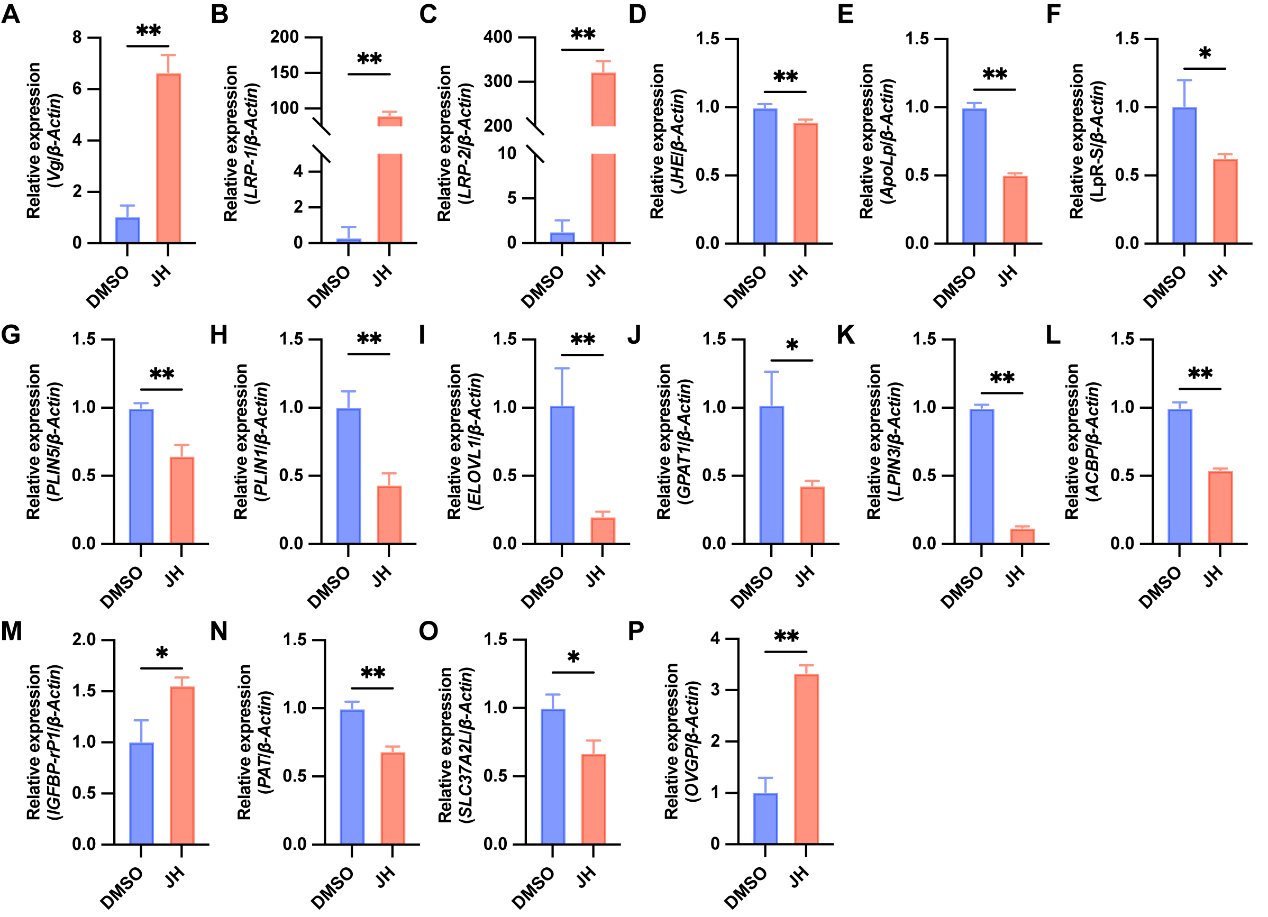


**Figure S12. qRT‑PCR validation of JH‑responsive gene expression in the fat body of adult locusts.** Total RNA was extracted from the fat body of 3‑day‑old adult females treated with JH III at 10 μg for 6 h., and gene expression was analyzed by qRT‑PCR. Relative expression levels were normalized to *β*‑*Actin*. DMSO, solvent control. *, *P* < 0.05; **, *P* < 0.01; ns, not significant; n = 3.


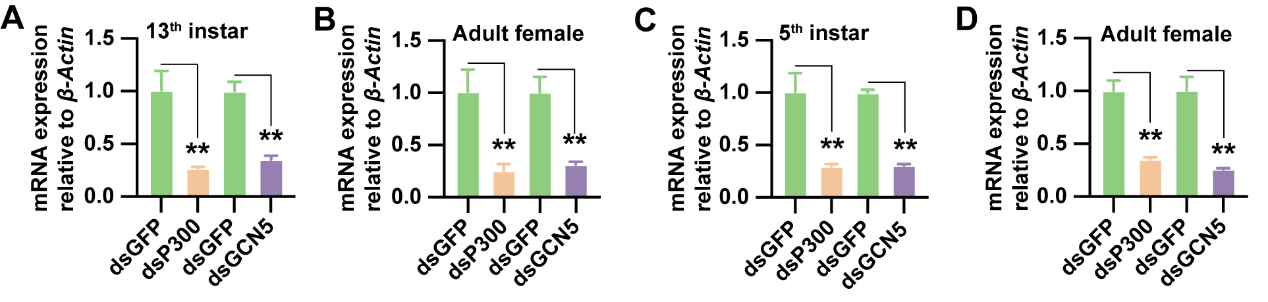


**Figure S13. *P300* and *GCN5* knockdown efficiency in the fat body** **of *P. americana* and** ***H. armigera*. A-B.** Knockdown efficiency of *P300* and *GCN5* in the fat body of penultimate 13^th^ instar nymph (*A*) and adult female cockroaches on day 6 (*B*). **, *P* < 0.01; n = 3. **C-D.** Knockdown efficiency of *P300* and *GCN5* in the fat body of penultimate 5^th^ instar larva (*C*) and adult female cotton bollworms on day 2 (*D*). **, *P* < 0.01; n = 3.


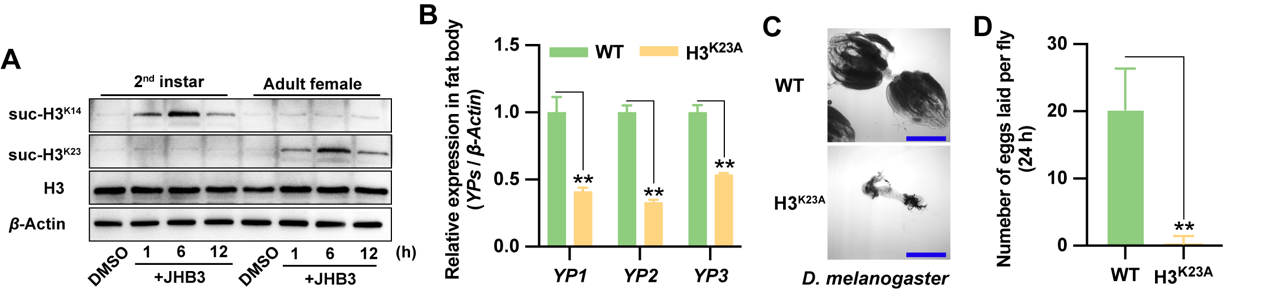


**Figure S14. Effect of H3^K23^ mutation on *Drosophila* reproduction. A.** Effect of JH on suc-H3^K14^ and suc-H3^K23^ in the fat body of penultimate 2^nd^ instar larvae and adult females of fruit flies treated with JHB3 for 1, 6 and 12 h. DMSO, solvent control. **B.** Relative expression levels of *yolk proteins* (*YPs*) in the fat body of 1-day-old H3^K23A^ mutant vs. wildtype (WT) adult female fruit flies. **, *P* < 0.01; n = 3. **C.** Representative ovarian phenotypes of H3^K23A^ mutant vs. wildtype (WT) adult females on day 2. Scale bars: 0.5 mm. **D.** Number of eggs laid by H3^K23A^ mutants vs. wildtypes (WT) 24 h post adult emergence. **, *P* < 0.01; n = 3.


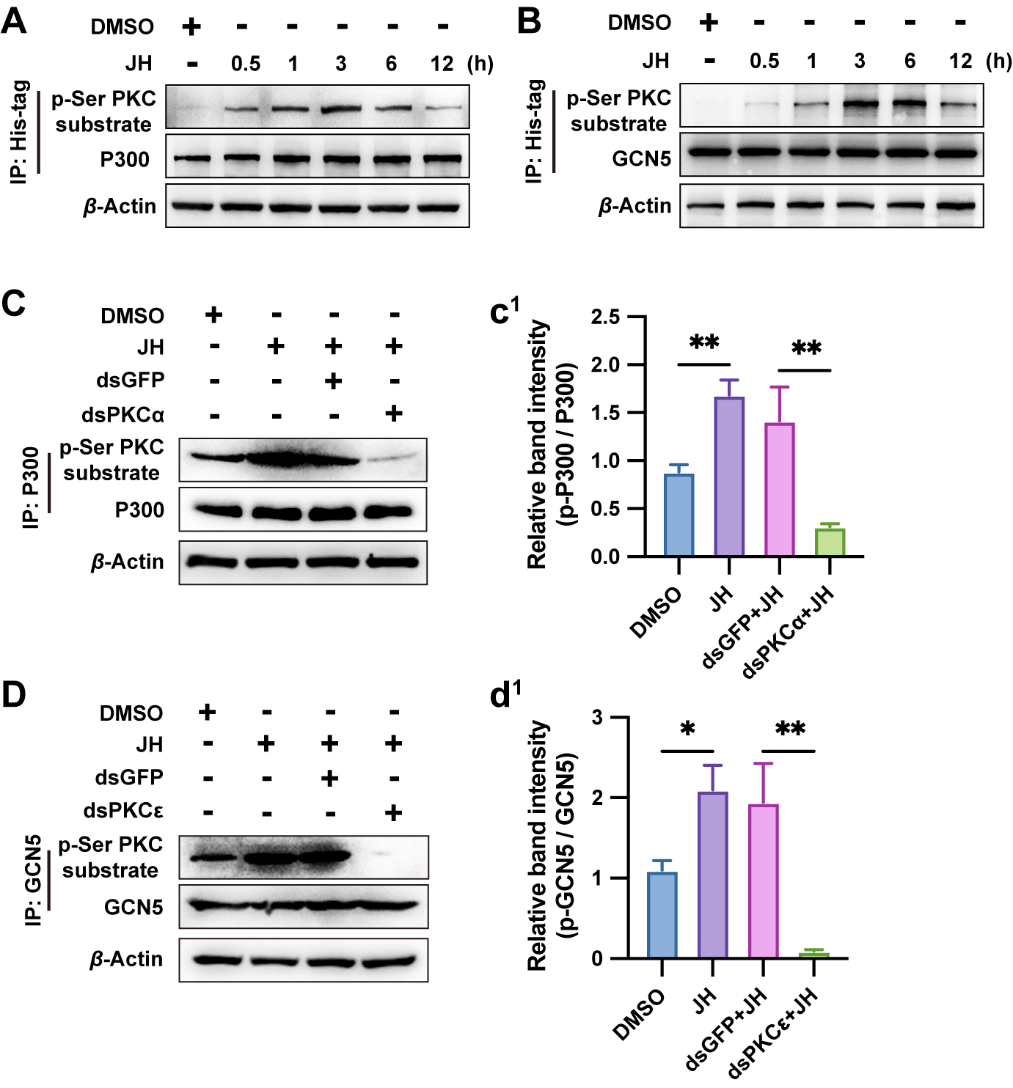


**Figure S15. JH-stimulated and PKC-mediated P300 and GCN5 phosphorylation.** (**A-B**) Time‑course analysis of JH effect on PKC-mediated phosphorylation of P300 (*A*) and GCN5 (*B*). Proteins were extracted from Sf9 cells transfected with pIEx-4-P300-RFP-His or pIEx-4-GCN5-RFP-His, followed by JH treatment. DMSO，solvent control. (**C-D**) Effect of *PKCα* on JH-induced P300 phosphorylation in nymphs (*C*) and *PKCε* knockdown on JH-induced GCN5 phosphorylation in adult locusts (*D*). Immunoprecipitation (IP) was carried out using antibodies against His-tag, P300 or GCN5, followed by western blot with a phospho‑(Ser) PKC substrate antibody. β‑Actin was used as a loading control. **c^1^ and d^1^.** Quantification of band intensities from three independent experiments of (*C*) and (*D*). *, *P* < 0.05; **, *P* < 0.01; n = 3.


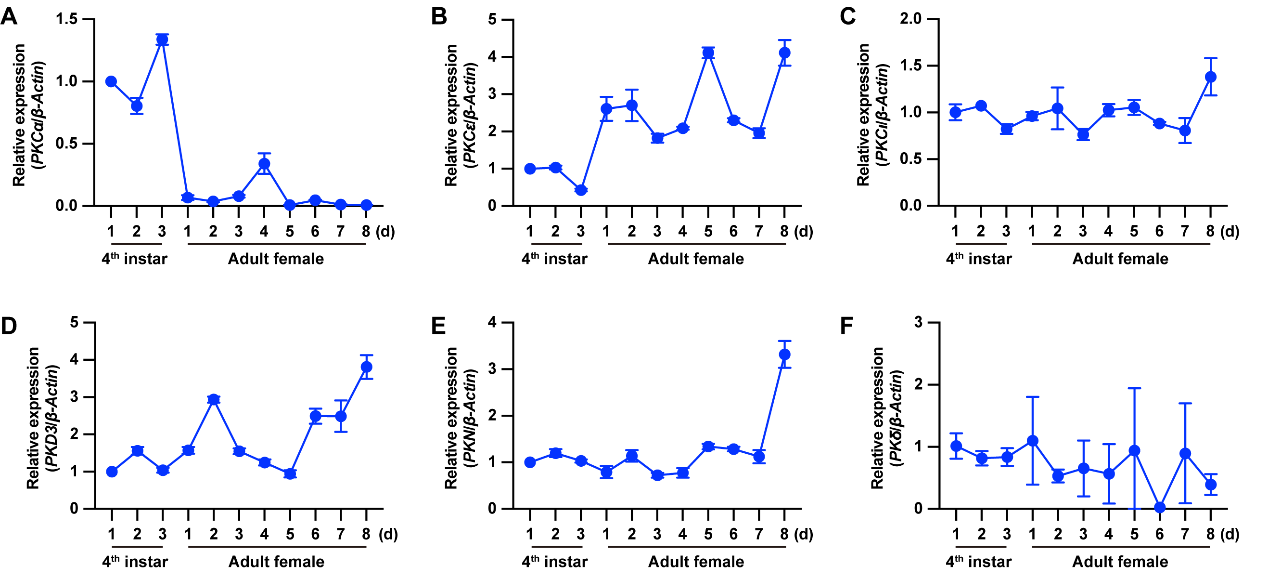


**Figure S16. Stage-specific expression profiles of PKC isoforms during locust nymphal and adult development.** qRT-PCR analysis of relative mRNA expression levels of *PKCα* (**A**), *PKCε* (**B**), *PKCι* (**C**), *PKD3* (**D**), *PKN* (**E**), and *PKCδ* (**F**) in the fat body of 4^th^ instar nymph and adult female locusts in the first gonadotropic cycle. n = 3.

**Table S1. Primers used for qRT-PCR, RNAi,** **vector construction, CRISPR/Cas9, and ChIP-qPCR**

| **qRT-PCR** | |
| --- | --- |
| **Primer** | **Oligonucleotide sequence (**5'-3'**)** |
| GCN5-qRT F | gcacagtaccagggctacatcaagg |
| GCN5-qRT R | atgctctcgatggggatggagcgga |
| P300-qRT F | gctcccagcaatcaaccaacggcat |
| P300-qRT R | ccctgaacttgagtgtgaggaggtg |
| HAT1-qRT F | ctgatgatgatcactggagaa |
| HAT1-qRT R | agattccaatagatgactgcc |
| CPT1A-qRT F | attgaaagggctgcctttatt |
| CPT1A-qRT R | tgtgacatgatagctgcatct |
| Ha-GCN5-qRT F | acatcaagctgccgcgggc |
| Ha-GCN5-qRT R | ggatgctgcggacgccttc |
| Ha-P300-qRT F | tgcagccacaaactgcaatc |
| Ha-P300-qRT R | cttcaaacaattatcacagcag |
| Ha-Vg-qRT F | aacattctcgtccgcatt |
| Ha-Vg-qRT R | cagcgtcacggaatatca |
| Pa-GCN5-qRT F | ggatcccgaattcaaacca |
| Pa-GCN5-qRT R | caggactgagagtcactgt |
| Pa-P300-qRT F | cctatgaatgcgataggtg |
| Pa-P300-qRT R | cacattgtgctgcactgaa |
| Pa-Vg-qRT F | tgctgatgaggacacaacct |
| Pa-Vg-qRT R | ccacttgcttcactggatcg |
| Krh1-qRT F | gcaagtcgttcggctacaa |
| Krh1-qRT R | gctgctccgagtggctctt |
| Vg-qRT F | cctacatcttagaaacgaaagtgac |
| Vg-qRT R | caacggacccctgtgatac |
| Met-qRT F | aatcacaaattcactctctccttca |
| Met-qRT R | atcggactgaataaaagtcgatcca |
| SRC-qRT F | tcccgcctaccacacttattccact |
| SRC-qRT R | tcgggccgtcgctcccaaccggact |
| Hairy-qRT F | gggtgctgataatagagcacaggag |
| Hairy-qRT R | ctgcaacagctgagacatcaactgg |
| Dm-YP1-qRT F | gaaccccatgagagtgctga |
| Dm-YP1-qRT R | aatgcctggttgacggagtt |
| Dm-YP2-qRT F | aggactttgagcagtacgcc |
| Dm-YP2-qRT R | acgttcacggtgttggtcag |
| Dm-YP3-qRT F | tacgcgatgctggatgttct |
| Dm-YP3-qRT R | cctggccgatcagatggatg |
| PKCα -qRT F | cggttgtggaacaagtgg |
| PKCα-qRT R | tggcttgaaaggaggttg |
| PKCδ-qRT F | ttgctgtacgggctatttc |
| PKCδ-qRT R | cagggcttccacgatgag |
| PKCε-qRT F | ccaagaatcctgccaaac |
| PKCε-qRT R | cagtgcttcccagtccat |
| PKCι-qRT F | ggcatgtgcaaggaaggt |
| PKCι-qRT R | gtgcccaccaatcaacacta |
| PKD3-qRT F | cgcctttatgtatccagc |
| PKD3-qRT R | ctgcttctccagttctcgt |
| PKN-qRT F | cagtgcgtatgaatcccg |
| PKN-qRT R | ccctggcaatgatgtctc |
| *β*-Actin-qRT F | aattaccattggtaacgagcgatt |
| *β*-Actin-qRT R | tgcttccatacccaggaatga |
| NCP14.9-qRT F | tcactgcctgagccaaaatg |
| NCP14.9-qRT R | cttggctgttttgcgagtttc |
| Abd-8-qRT F | atcgcagtcctgatgatggt |
| Abd-8-qRT R | cccgtctcgtagctccatt |
| CP-qRT F | tacaacgccatcccgcagta |
| CP-qRT R | agcctagctcttgttgaaggtg |
| obst-E-like-qRT F | actgagacggagaaacaacga |
| obst-E-like-qRT R | gggcgaggaggaggtttta |
| Obst-E2-qRT F | ttgtgttgctggtgttggat |
| Obst-E2-qRT R | tatctgccgtttggttcagga |
| obst-E1-qRT F | caaatgcgacgtcttctgga |
| obst-E1-qRT R | ttgaatccgttcgccacttc |
| Tret1-L-qRT F | tcaggtccatcacttgcatca |
| Tret1-L-qRT R | ccacactgggctttcaggta |
| Tret1-2H1-qRT F | tcttctacacgcagggcatc |
| Tret1-2H1-qRT R | agaagacgagcagcacctt |
| BR-qRT F | catcgggcgatcaatcacag |
| BR-qRT R | gcgacggtttaccagaagga |
| Grh-qRT F | aagctggacgagttgtacca |
| Grh-qRT R | tgtgtcgtggttgtagaagc |
| cwo-qRT F | gctcttcaccaggttcagga |
| cwo-qRT R | cccgcaactgactacatctg |
| Sox-2-qRT F | ccaacaacaacagcaccaac |
| Sox-2-qRT R | cggtcgacgttctggttctt |
| CP18.7-L-qRT F | atcccagccatcgccaat |
| CP18.7-L-qRT R | tggtatcacagtagtcgagtca |
| HEXDL-qRT F | acgtgcggcacaacttct |
| HEXDL-qRT R | ccacccactctgtgattgtg |
| Tret1-2H2-qRT F | tcttcgtcgtcgccttctc |
| Tret1-2H2-qRT R | caaacaccttcgtcaccgtaa |
| E78C-qRT F | taccactacggggtcacctc |
| E78C-qRT R | tttcttgaagcggcagtactg |
| E74-qRT F | gcccaagaagcttcgtactg |
| E74-qRT R | acacccttttccctgtttgtc |
| HR38-qRT F | atcagcgaggcagagaagg |
| HR38-qRT R | gagctctaaggaagcggact |
| FoxN3-qRT F | cctccctcaacgacttcctc |
| FoxN3-qRT R | gttgtagaggatgcgatggg |
| ILP1-qRT F | agtccgacctgttcctcct |
| ILP1-qRT R | tcttgtgaagccttcttgaaca |
| MMP2L-qRT F | acctgcaacaccaactacga |
| MMP2L-qRT R | gtttcttggcaggctgtacc |
| CASP1-qRT F | ttgtcatggctgtgctgtc |
| CASP1-qRT R | ggcacgcctgaatgaagaat |
| CASP1L-qRT F | gatcgtgggtggtgtcattg |
| CASP1L-qRT R | gcttcagacccggatcgat |
| CHT2-qRT F | tggatggaatgagggctctg |
| CHT2-qRT R | cacctctttgcgtcggatatt |
| ApoLp-qRT R | tcgaggtccttcaagtttgc |
| ApoLp-qRT F | accaggtacacagggtgaac |
| ELOVL1-qRT R | tccattgctgacaggtgagt |
| ELOVL1-qRT F | tgtgtggtggattggaatgc |
| OVGP-qRT R | ccacctctttgcgtcggata |
| OVGP-qRT F | tggatggaatgagggctctg |
| LRP-2-qRT R | gcccttctccatcaacagga |
| LRP-2-qRT F | tggatggtactggcaagagg |
| LRP-1-qRT R | tgcccttctccatcaacagg |
| LRP-1-qRT F | ggatggaactggcaaaaggg |
| CHS2-qRT R | atagacaccagggcacgtt |
| CHS2-qRT F | agattccggcgatgatgact |
| LpR-S-qRT R | tctcaggagcacaggttgtt |
| LpR-S-qRT F | agaaggcgtgcaatgaaact |
| ACBP-qRT R | tgcatcccattttgctttgc |
| ACBP-qRT F | cagctgaagatgtcaagaagct |
| PAT-qRT R | gatggctagcacgacgaaat |
| PAT-qRT F | ctggtgaagtgctcccaaga |
| GPAT1-qRT R | aacaaggccaggacctagaa |
| GPAT1-qRT F | tttaggaccggtgctactgt |
| JHE-qRT R | caggtgtagggttcccagtt |
| JHE-qRT F | aaggtgcttgccatggaga |
| SCD-qRT R | ttgtggggatcagcatttgtg |
| SCD-qRT F | ttggtcccatcgttcctacc |
| PLIN1-qRT R | accagcatccttcaccgtag |
| PLIN1-qRT F | acggaactctttgcaaaggtc |
| PLIN5-qRT R | tgagcaaaatccagcagcttt |
| PLIN5-qRT F | ccaccaacgacacttgaaca |
| IGFBP-rP1-qRT R | ctttcacaaggtccccaatgtt |
| IGFBP-rP1-qRT F | gccagagtctttgtgcatgt |
| LPIN3-qRT R | gcctgttgacctcgtactct |
| LPIN3-qRT F | atttggccgagctttcaagg |
| SLC37A2L-qRT F | tattgctggtggtggtttcc |
| SLC37A2L-qRT R | tctggtgctgatcctttggt |
| **RNAi** | |
| **Primer** | **Oligonucleotide sequence (**5'-3'**)** |
| GCN5-RNAi F | gcgtaatacgactcactataggggccagaaggagatcgtgaag |
| GCN5-RNAi R | gcgtaatacgactcactatagggaccggtagtactccgtgtcg |
| P300-RNAi F | gcgtaatacgactcactatagggagcttgttttgctcctgcat |
| P300-RNAi R | gcgtaatacgactcactatagggccaacaccaagaccaggagt |
| CPT1A-RNAi F | gcgtaatacgactcactatagggcgttacttcaaggtgcccat |
| CPT1A-RNAi R | gcgtaatacgactcactataggggatcgtagcctttcccatga |
| HAT1-RNAi F | gcgtaatacgactcactatagggtgttcggaagccagaagact |
| HAT1-RNAi R | gcgtaatacgactcactatagggtacggggcctgatattcttg |
| Ha-GCN5-RNAi F | gcgtaatacgactcactatagggctctcatcaaggaaggcagg |
| Ha-GCN5-RNAi R | gcgtaatacgactcactatagggacgctggtgaacatggtgta |
| Ha-P300-RNAi F | gcgtaatacgactcactatagggaattattccgtcaggacccc |
| Ha-P300-RNAi R | gcgtaatacgactcactataggggcttcctcccacaatccata |
| Pa-GCN5-RNAi F | gcgtaatacgactcactatagggccattgaaggtcctcttgga |
| Pa-GCN5-RNAi R | gcgtaatacgactcactatagggccatggcaccagtaggaagt |
| Pa-P300-RNAi F | gcgtaatacgactcactataggggtttacagtcaccgccgaat |
| Pa-P300-RNAi R | gcgtaatacgactcactatagggaccaccagtcgcattacctc |
| PKCα-RNAi F | gcgtaatacgactcactatagggtctttaagcagccgacgttt |
| PKCα-RNAi R | gcgtaatacgactcactatagggtcggcctcttctttcagtgt |
| PKCδ-RNAi F | gcgtaatacgactcactatagggaaagacgttacggaacaccg |
| PKCδ-RNAi R | gcgtaatacgactcactataggggcactttctgtggcagttga |
| PKCε-RNAi F | gcgtaatacgactcactatagggagcagtggaggaggtagcaa |
| PKCε-RNAi R | gcgtaatacgactcactataggggctgcaagggctagaatacg |
| PKCι-RNAi F | gcgtaatacgactcactatagggagaaggcgcttgtcactgat |
| PKCι-RNAi R | gcgtaatacgactcactatagggtgcccaccaatcaacactaa |
| PKD3-RNAi F | gcgtaatacgactcactatagggtgttccgtcatgaccacagt |
| PKD3-RNAi R | gcgtaatacgactcactatagggcggcactgtaacccttgttt |
| PKN-RNAi F | gcgtaatacgactcactataggggttcacaacaagcctgggat |
| PKN-RNAi R | gcgtaatacgactcactatagggcttcctcttcccagcaactg |
| GFP-RNAi F | gcgtaatacgactcactataggtggtcccaattctcgtggaac |
| GFP-RNAi R | gcgtaatacgactcactataggcttgaagttgaccttgatgcc |
| **Vector construction** | |
| **Primer** | **Oligonucleotide sequence (**5'-3'**)** |
| **Recombinant pIEx-4-RFP-His vector construction** | |
| P300-OE F | aacaaccaagtgaccatggggattgctgctggtgcactt |
| P300-OE R | ggtgatggtgatggtggtgctggactgaacctgctcca |
| GCN5-OE F | aacaaccaagtgaccatgagccagcagacactgggg |
| GCN5-OE R | gacgtcctcggaggatttgtcccagaggccgagct |
| **CRISPR/Cas9** | |
| **Primer** | **Oligonucleotide sequence (**5'-3'**)** |
| H3K14A-sgRNA-F | taatacgactcactatagccgcaagtccaccggc |
| H3K14A-sgRNA-R | ttctagctctaaaacttccgccggtggacttgcg |
| H3K23A-sgRNA-F | taatacgactcactatagaggaagagcgcgcccg |
| H3K23A-sgRNA-R | ttctagctctaaaacgtggcgggcgcgctcttcc |
| H3K14A-donor-F | atggcccgcacaaaccaaacagcacgaaaatcaaccggcggagcagcgccgcgcaaacagctcgccaccaaggcggcgaggaagagcgcacccgccacc |
| H3K14A-donor-R | ggtggcgggtgcgctcttcctcgccgccttggtggcgagctgtttgcgcggcgctgctccgccggttgattttcgtgctgtttggtttgtgcgggccat |
| H3K23A-donor-F | cgcaagtccaccggcggcaaagcaccacggaagcaactcgccaccgcagcggcgaggaagagcgcgcccgccaccggcggcgtcaacaagccccaccgc |
| H3K23A-donor-R | gcggtggggcttgttgacgccgccggtggcgggcgcgctcttcctcgccgctgcggtggcgagttgcttccgtggtgctttgccgccggtggacttgcg |
| H3K14A-seq-F | aaaccaaacagcacgaaaatca |
| H3K14A-seq-R | tctctcgcacgaggcgct |
| H3K23A-seq-F | cggcaaagcaccacggaagcaa |
| H3K23A-seq-R | tctctcgcacgaggcgct |
| **ChIP-qPCR** | |
| **Primer** | **Oligonucleotide sequence (**5'-3'**)** |
| NCP14.9-chip-F | taattggcgcggaaatgtagc |
| NCP14.9-chip-R | tcacccgcatacaactaaacg |
| obst-E-like-chip-R | aagcgaaaaggcagtcagtc |
| obst-E-like-chip-F | tcccttgcaccttaattctgtc |
| E74-chip-R | ggtacggcagcgcataaaat |
| E74-chip-F | ccgagcgctacaccttcat |
| Tret1-L-chip-R | gccactgacgccattgtaaa |
| Tret1-L-chip-F | tccagatccaaggaagacact |
| obst-E1-chip-R | taacgcctggacaccaaga |
| obst-E1-chip-F | ccgattgaatagctgcctgaa |
| OVGP-chip-R | tagccgagcggtctaaagc |
| OVGP-chip-F | gtggtcatcagtccgtagct |
| FoxN3-chip-R | gccatatcctctgaaccatgtg |
| FoxN3-chip-F | ctcctaaaccattggaccgatt |
| Abd-8-chip-R | agctgaaaatgactgacaacct |
| Abd-8-chip-F | aatagcgcatgggaagaaagaa |
| ILP1-chip-R | tcgctaaaatgaagccatccc |
| ILP1-chip-F | gggcaacttacgaaccagtaa |
| Grh-chip-R | tctcgcatgcgccaaacaa |
| Grh-chip-F | actgtgctgtgacgtcatcc |
| HR38-chip-R | caacacttccctgggaacg |
| HR38-chip-F | taaccggctgcactgaagta |
| HEXDL-chip-R | tcacttcgctactgctttgaga |
| HEXDL-chip-F | gccaagcgtcgcaaatataatt |
| E78C-chip-R | cattactgccgtgcacatgt |
| E78C-chip-F | aggtggatgtgttggacgta |
| CP18.7-L-chip-R | ggccggcatgacttaactc |
| CP18.7-L-chip-F | gcatatgctacgttcgaatgc |
| Tret1-2H2-chip-R | gtcagagatggagcctttgc |
| Tret1-2H2-chip-F | caagatggcgcagtggtaa |
| Tret1-2H1-chip-R | caccctgaccaatacaacaca |
| Tret1-2H1-chip-F | ttcctgaaagtttgaccgtacc |
| BR-chip-R | ggtggcccctctttctccta |
| BR-chip-F | gcacaaagcgccgttaact |
| Sox-2-chip-R | ccgcggctacagatttattcag |
| Sox-2-chip-F | ttcggaacatatgtcgtgtctc |
| CP-chip-R | tggttgaagcttacgtgtacaa |
| CP-chip-F | tgagagtcgataaaccccgtaa |
| CASP1L-chip-R | tctggcagcacaaaacatct |
| CASP1L-chip-F | atctctggtcgggtgttcc |
| CASP1-chip-R | ccccagcctatctttcagga |
| CASP1-chip-F | cgccaccacaagaaaagaga |
| MMP2L-chip-R | gcacccgggccttgttaat |
| MMP2L-chip-F | aggaactgctgcaggaagt |
| cwo-chip-R | gagggaagtgtgtggggtaa |
| cwo-chip-F | actggtttgatgcagctctc |
| Obst-E2-chip-R | gctcttcgttcgaccccat |
| Obst-E2-chip-F | gcaccacctcacttttcact |
| ApoLp-chip-R | tgtgtgtgtgtgtgcattgt |
| ApoLp-chip-F | agttccaggtgtttcttgaagg |
| ELOVL1-chip-R | cagagacctgcaccttcca |
| ELOVL1-chip-F | aatgcaccgggtactcagtt |
| VgA-chip-R | gtgatgcccacgagtaggaa |
| VgA-chip-F | gctcacaactgtccttacatgt |
| OVGP-chip-R | gcttctgccagtggatcatc |
| OVGP-chip-F | ggccgactcttgagtattgc |
| LRP-1-chip-R | ggaggtgacactggcactag |
| LRP-1-chip-F | tctcaccacctgaaggctag |
| LRP-2-chip-R | ggaggtggagcacaaaatcc |
| LRP-2-chip-F | ccagaaggaaggaaaggaactg |
| CHS2-chip-F | acacccatactcacacagaca |
| CHS2-chip-R | tgtattctgtggggtggttct |
| LpR-S-chip-R | gtgtgccgtaagtaccaaca |
| LpR-S-chip-F | gacaaggacggtatgcgatt |
| ACBP-chip-R | cccgattgagcatgtctgag |
| ACBP-chip-F | ccgtattgcgctcctgaatc |
| PAT-chip-R | catcacgttcccgactttga |
| PAT-chip-F | gacatgtattgccctgctgaa |
| GPAT1-chip-R | gacatgtattgccctgctgaa |
| GPAT1-chip-F | ttccaatccccgaccgtaat |
| JHE-chip-R | tgtggcggagggtgcttta |
| JHE-chip-F | atgctacccacgcgtaatca |
| SCD-chip-R | gtgacgccatgcacagaa |
| SCD-chip-F | gagtagccgagcggtctaa |
| PLIN1-chip-R | gctgtctcacgaagcactg |
| PLIN1-chip-F | cgtgtgagcgtgggtattga |
| PLIN5-chip-R | tagttcttggaccgcgctta |
| PLIN5-chip-F | cacccagatgcacttctcag |
| IGFBP-rP1-chip-R | ctgaccaatccacggttttct |
| IGFBP-rP1-chip-F | ggacggatcgctagaaacag |
| LPIN3-chip-R | accacatgtcaatttcgcaatg |
| LPIN3-chip-F | tgtgtgtgtgtgtgcattgt |
| CHS2-chip-R | caattgtgcagctgtgggta |
| CHS2-chip-F | tgggtgtggggaaatgtga |
| SLC37A2L-chip-R | cgactttagctgcactaggg |
| SLC37A2L-chip-F | tgatgaccacgcacatttaca |

Note: Ha, the cotton bollworm *Helicoverpa armigera*; Pa, the American cockroach *Periplaneta americana*; Dm, the fruit fly *Drosophila melanogaster*.
